# Assessment of the Binding Patterns for Endocrine Disrupting Chemicals in Complex with Estrogen and Androgen Receptors by Leveraging the Asclepios Enalos KNIME Nodes

**DOI:** 10.1021/acs.jcim.5c01437

**Published:** 2025-10-20

**Authors:** Haralampos Tzoupis, Michail Papadourakis, Konstantinos D. Papavasileiou, Oliver Burk, Volker M. Lauschke, Andreas Tsoumanis, Georgia Melagraki, Antreas Afantitis

**Affiliations:** 1 Department of ChemoInformatics, 443801 NovaMechanics Ltd., Nicosia CY-1070, Cyprus; 2 Department of ChemoInformatics, NovaMechanics MIKE., Piraeus GR-185 45, Greece; 3 Dr Margarete Fischer-Bosch Institute of Clinical Pharmacology, Stuttgart 70376, Germany; 4 University of Tübingen, Tübingen 72074, Germany; 5 Department of Physiology and Pharmacology and Center for Molecular Medicine, Karolinska Institutet and University Hospital, Stockholm 17177, Sweden; 6 Department of Pharmacy, the Second Xiangya Hospital, Central South University, 139 Renmin, Changsha 410017, China; 7 Division of Physical Sciences and Applications, Hellenic Military Academy, Vari 16672, Greece; 8 Division of Data Driven Innovation, Entelos Institute, Larnaca CY-6059, Cyprus

## Abstract

Endocrine disrupting chemicals (EDCs) have been shown to mediate metabolic disruptions in human cells and have been associated with severe adverse health effects. By antagonizing the hormones that act on nuclear hormone receptors, like the estrogen receptor α (ERα) and the androgen receptor (AR), these chemicals disrupt the regulation of various biochemical processes, thereby adversely affecting metabolic homeostasis. The expression of estrogen and androgen receptors in the liver and pancreas, which play an important role in lipid and glucose homeostasis regulation, has made them prime targets affected by EDCs. The different chemical structures of EDCs impose limitations on elucidating their binding mechanisms in nuclear receptors. In this context, *in silico* tools are able to highlight the potential interactions between the chemicals and the receptors. The aim of this study is to apply molecular simulation and experimental techniques to identify common patterns in the binding process of selected EDCs to ERα and AR and, thus, pinpoint key elements that could be characterized as molecular initiating events (MIE). MM-GBSA and alchemical relative binding free energy (RBFE) calculations have verified the trends observed in the experimental assays regarding the binding affinity of bisphenol compounds. The findings that confirm the agreement between computational and experimental methods offer a framework for future studies on the behavior of EDCs with other metabolically relevant receptors.

## Graphical abstract

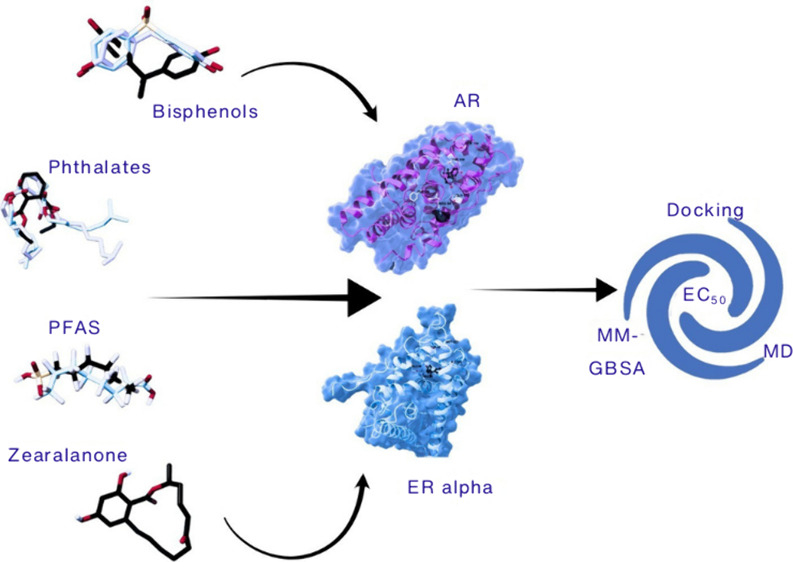

## Introduction

Endocrine disrupting chemicals (EDCs) are defined as “exogenous substances that induce adverse health effects in intact organisms”.^1,2^ EDCs bind to various protein targets inside the cells and cause disruptions in metabolic pathways. As their name implies, EDCs exert their action mainly by affecting hormone synthesis and the homeostasis of the endocrine system.^3^ These chemicals have also been linked to reproductive system dysfunction and developmental disorders in humans.^4^ The most common pathway for EDCs to exert their function is through binding to nuclear hormone receptors such as estrogen and androgen receptors.^5^ The binding is facilitated by their diverse chemical structures and their molecular properties, particularly their structural similarities with endogenous ligands such as steroid hormones.^5^ Upon binding to hormone receptors, EDCs can cause alterations in their functionality and inhibit or activate hormonal responses.^6^

Although EDCs have initially been reported to act mainly through interactions with the estrogen and androgen receptors, recent research has shown that they exert diverse actions through interactions with receptors like the peroxisome proliferator-activated receptor γ (PPARγ)^7^ and the pregnane X receptor (PXR).^8^ The chemical differences observed for the various EDCs mirror the variability in their action and show that they can have pleiotropic effects. For instance, dichloro-diphenyl-trichloroethane (DDT) can have antagonistic action against androgens through its metabolites.^9–12^

The estrogen receptor α (ERα) is primarily expressed in the reproductive tissues, white adipose tissue, liver, and breast,^13^ while the androgen receptor (AR) has been shown to have an important role in various systems like the reproductive, cardiovascular, immune, or nervous system.^14,15^ Both receptors belong to the same family of proteins and, thus, share structural characteristics that reflect common functional and regulatory aspects. The common functional motif of these receptors comprises two distinct areas, the ligand binding domain (LBD) and the DNA-binding domain (DBD), that are separated by a hinge region.^16–18^ Estrogens in the liver can have dual effects either by promoting or inhibiting liver functions^19^ and cancer development.^20^ Moreover, estrogens have been associated with glucose metabolism and pancreatic regulation of glycemic control.^21^ Similarly, the AR has been associated with cancer development,^22^ as well as other liver^23^ and pancreatic disorders.^24,25^

The diverse functionality reported for these two receptors has made them the primary focus as biomarkers for EDC risk assessment. Binding aspects of different groups of EDCs, such as PFAS,^26,27^ phthalates,^28,29^ or bisphenols,^30–32^ have been studied via the implementation of computational approaches. The results identified possible aspects of the binding mechanism for these chemicals, highlighting various structural components of the receptors.

Several molecular dynamics (MD) studies have been previously performed in an effort to advance our understanding of how small molecules interact with ERα and AR,^33^ focusing specifically on their LBDs. For ERα, early work by McGee et al.^34^ demonstrated how removal of antagonists affects receptor conformation, while later studies such as those by Ng^35^ and Puranik et al.^36^ highlighted ligand-specific fluctuations, particularly in helix 12 (H12), which is crucial for activation.^37^ More recent work by Sinyania et al.^38^ investigated flavonoids to reveal how structural dynamics and binding energetics govern receptor modulation. On the other hand, AR-focused studies have shown that agonists and antagonists differentially influence AR flexibility and coactivator binding. Wahl and Smieško^39^ addressed the challenge of lacking antagonist-bound crystal structures for the AR by employing MD simulations to generate antagonist conformations. This approach improved the virtual screening accuracy for AR antagonists and highlighted the role of H12 dynamics in distinguishing between agonist and antagonist binding modes. Gim et al.^40^ and Liu et al.^41^ used MD to elucidate how ligand binding regulates AR activation and coregulator interactions. Additionally, efforts by Xu et al.^42^ extended the analysis to DNA-binding behavior and dimerization, emphasizing the structural intricacies underlying AR function.

With respect to EDCs, a comprehensive study by Tan et al.^43^ analyzed over 4000 compounds using molecular docking and MD simulations to identify structural fragments responsible for binding and activation of ERα. They discovered that primary and secondary EDC fragments facilitate receptor binding, while tertiary fragments determine the activity type (agonist, antagonist, or mixed). Additionally, a QM/MM study focused on bisphenol A (BPA) demonstrated that BPA binds to ERα’s active site similarly to 17β-estradiol, leading to conformational changes associated with estrogenic activity.^44^ Similarly, Huang et al.^45^ explored the potential binding mechanism of bisphenol A to AR, while a recent study by Pathak et al.^46^ explored the binding mechanics of other bisphenol analogues.

The scope of the present study was to employ molecular docking calculations, MD simulations, molecular mechanics-generalized born surface area (MM-GBSA) analysis, and alchemical relative binding free energy calculations methods to investigate and compare the binding patterns of common EDCs with diverse chemical structures from different chemical groups (e.g., bisphenols, phthalates, and PFAS). The simulations provide the necessary information for identification of potential common binding patterns between the different EDCs. They were corroborated by experimental assays employing ERα and AR reporter gene constructs in transfected HEK293 cells, allowing validation of both the binding activity and functional response. Consequently, this information can be employed to identify molecular initiating events (MIEs) and, in combination with *in vivo* and *in vitro* studies, assist in developing a reliable EDC risk assessment tool.

## Materials and Methods

### Protein and Compound Structure Preparation

The crystal structures of (i) the estrogen receptor α in complex with the drug tamoxifen (PDB ID: 3ert)^17^ and (ii) the androgen receptor in complex with 5α-dihydrotestosterone (PDB ID: 1t7t)^18^ were employed for the molecular docking and molecular dynamics simulations. The preparation of the protein structures was performed with the AsclepiosPDBFixer KNIME node (Figure 1A). The A chain of the receptors was retained, and all heteroatoms were removed, followed by the addition of hydrogen atoms at neutral pH (i.e., pH = 7.4). All protein residues in the files have been renumbered starting from the first residue.

**Figure 1:**
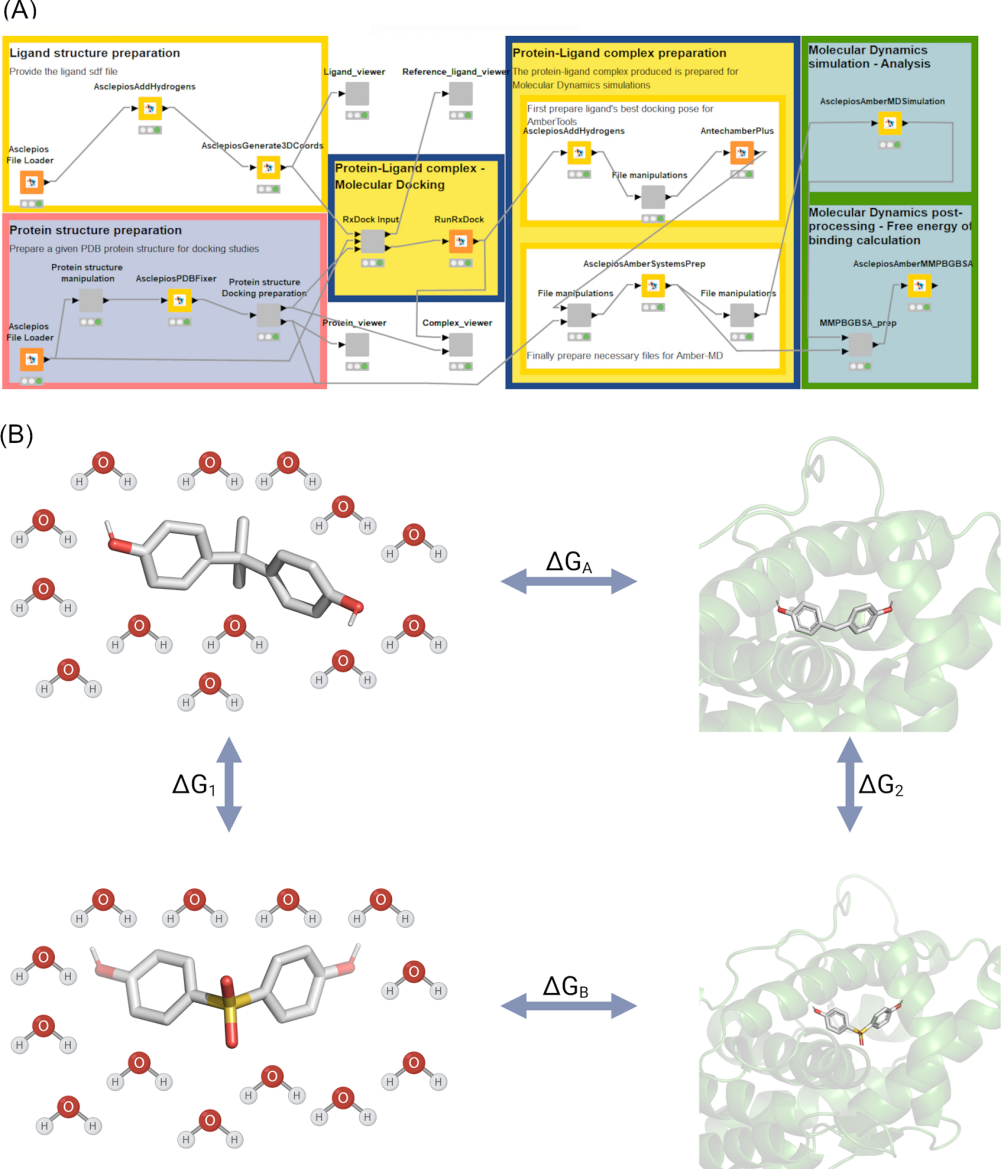
(A) Enalos Asclepios KNIME workflow for the preparation and execution of the molecular docking performed in this study with Autodock Vina and molecular dynamics (MD) simulations. (B) Schematic representation of the thermodynamic cycle for the perturbation of ligand A (bisphenol A) to ligand B (bisphenol S). The relative binding free energy (ΔΔ*G*_bind_) of bisphenol S with respect to bisphenol A can be calculated either directly (horizontal processes, Δ*G*_B_–Δ*G*_A_) or via an alchemical path (vertical processes, Δ*G*_2_–Δ*G*_1_).

The structures of the EDCs studied (Table 1) were downloaded from the PubChem database.^47^ Open Babel^48^ was employed for the conversion of the chemical structures to the SDF format. The hydrogen atoms were added at pH 7.4, using the AsclepiosAddHydrogen node, and the 2D structures were converted into low-energy 3D conformations by implementing the AsclepiosGenerate3Dcoordinates node (Figure 1A). All the nodes required for ligand and protein structure preparation are incorporated in the Enalos Asclepios KNIME platform (Figure 1A).^49–51^

**Table 1:**
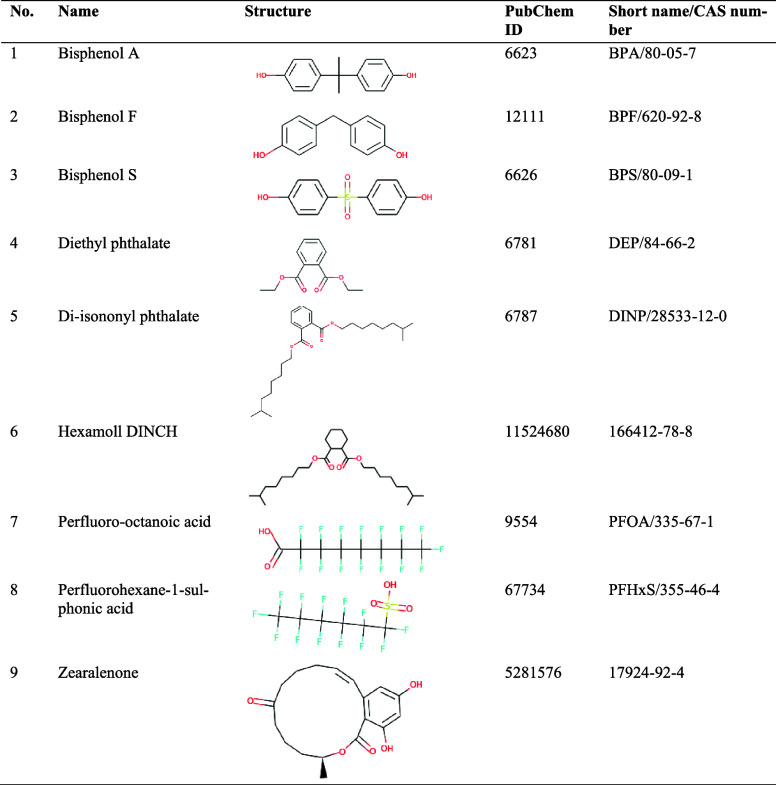
Endocrine Disrupting Chemicals Employed in the Present Study

### Molecular Docking Simulations

All simulations were performed using Autodock Vina^52^ software as implemented in the Enalos Asclepios KNIME nodes (Figure 1A). For ERα, the coordinates (*x*, *y*, and *z*) of the active site were 30.2, −2.9, and 23.4 and the box size was set at 21 Å × 24.5 Å × 19.5 Å, while for AR, the coordinates were −0.9, 3.1, and 36.9 and the box size was 31 Å × 24 Å × 15 Å, respectively. Detailed information is available in the Supporting Information. To assess the validity of the docking protocol, we have isolated the crystal structures of tamoxifen and 5α-dihydrotestosterone, respectively, and redocked the compounds in ERα and AR following the same protocol.

### Molecular Dynamics (MD) Simulations

The preparation of the protein-EDC complexes was performed using the workflow depicted in Figure 1A. The best scoring docking conformation of each compound was employed as the initial conformation for the MD simulations. All simulations were performed with the OpenMM 7.5 software^53^ as part of the Asclepios KNIME workflow. The AntechamberPlus and AsclepiosAmberSystemsPrep nodes, implemented in the Enalos Asclepios KNIME nodes, were used for constructing the parameters of each protein-compound complex. The AMBER14SB^54^ and the Generalized Amber Force Field (GAFF)^55^ were employed for the building of the parameters for the protein and respective ligands. All protein-EDC complexes were solvated in water, using the SPC/E^56,57^ model and a truncated octahedron solvent box with a 10 Å buffer distance. The SPC/E model has been reported to better reproduce bulk water properties.^58,59^ Periodic boundary conditions were applied in the system, and the total charge of the systems was neutralized by adding the respective number of Na^+^/Cl^–^ ions.

The systems were subjected to energy minimization with positional restraints on the proteins and ligands by using a harmonic force constant that was gradually reduced until fully abolished. The heating of the system was performed to the target temperature of 300 K for 200 ps, in the canonical ensemble (NVT), using the Langevin thermostat,^60^ followed by pressure equilibration at 1 atm in isobaric–isothermal conditions (NPT ensemble). Finally, the production run was performed for 200 ns (3 independent runs for each complex), with no positional restraints imposed on the proteins and ligands in the NPT ensemble.

### Molecular Mechanics-Generalized Born Surface Area Analysis (MM-GBSA)

The estimation of the relative ligand binding affinities was performed using the MM-GBSA methodology. MM-GBSA has been shown to perform well in predicting relative binding affinities, despite not always being able to reproduce the experimental results correctly.^61^ The calculations for the protein complexes were conducted with the AsclepiosAmberMMPBGBSA node, using the Enalos Asclepios implementation of the AmberTools21 MM-GBSA.py script^62^ of the respective KNIME workflow node over the 1000 last trajectory frames. All figures were prepared using the PyMOL Open-Source software.^63^

### Alchemical Relative Binding Free Energy Calculations

A nonequilibrium free energy workflow^64–66^ was employed to calculate the relative binding free energies of bisphenol F to ERα and AR, in comparison with bisphenols S and A. An example of the thermodynamic cycle applied in this study is shown in Figure 1B. The parameters for the proteins and the ligands were built using the same method described above. Following ligand parametrization, hybrid structures and topologies for each ligand pair were generated using the pmx^67^ module of GROMACS 2024.4.^68^ Simulation systems for both the solvated ligands and the ligand–protein complexes were prepared by placing the molecules in cubic boxes, ensuring a minimum distance of 15 Å between the solute and the box boundaries.

For each ligand pair, simulations were carried out for both physical end states—state A and state B—corresponding to ligands 1 and 2, respectively. The systems were first subjected to energy minimization, followed by a 500 ps equilibration in the NVT ensemble at 310 K. This was followed by a 10 ns production run in the NPT ensemble at 310 K and a pressure of 1 bar.

Temperature control in the simulations was achieved using Langevin dynamics^69^ with a collision frequency of 1 ps^–^^1^. Pressure was maintained at 1 bar using the Parrinello–Rahman barostat,^70^ with a time constant of 2 ps and a compressibility of 4.5 × 10^–^^5^ bar^–^^1^. All bonds involving hydrogen atoms were constrained using the LINCS algorithm.^71^ Long-range electrostatic interactions were handled using the Particle Mesh Ewald (PME) method,^72^ with a real-space cutoff of 12 Å, a Fourier grid spacing of 1 Å, and a relative interaction strength at the cutoff set to 10^–^^6^. Short range electrostatic and van der Waals interactions were calculated with a cutoff of 12 Å and a switching distance of 10 Å. For each protein–ligand complex, the nonequilibrium workflow was performed in triplicate, and the reported binding free energies (Δ*G*_bind average_) represent the mean across the replicates. Statistical uncertainties are provided by propagating the errors of the respective calculations.

The detailed protocols employed for the MD simulations, MM-GBSA, and alchemical free energy calculations are described extensively in the Supporting Information.

### Chemicals and Reagents

DMSO, bisphenol A (BPA), bisphenol F (BPF), and bisphenol S (BPS) were purchased from Sigma-Aldrich (Taufkirchen, Germany). 11-Ketodihydrotestosterone (11-ketoDHT) was obtained from MedChem Tronica (Sollentuna, Sweden). Compounds for chemical treatment of transfected cells were prepared as 1000× stocks in DMSO. Dulbecco’s modified Eagle’s medium (DMEM) was provided by Thermo Fisher Scientific (Waltham, MA, USA). l-Glutamine, sodium pyruvate, and penicillin–streptomycin mixture were provided by Biozym (Hessisch Oldendorf, Germany). Fetal bovine serum (FBS) was purchased from Sigma-Aldrich. PEI MAX transfection-grade linear polyethylenimine hydrochloride MW 40,000 (PEI MAX 40K) was obtained from Polysciences (Warrington, PA, USA).

### Cell Culture

HEK293 cells (ACC305, DSMZ, Braunschweig, Germany) were cultivated at 37 °C in 5% CO_2_ in DMEM supplemented with 10% FBS, 2 mM l-glutamine, 1 mM sodium pyruvate, 100 U/mL penicillin, and 100 μg/mL streptomycin. For exposure studies, cells were cultivated in phenol red-free DMEM, supplemented with 1% dextran-coated charcoal-treated FBS.

### Plasmids

Part of the open reading frame (NM_000125) of human ERα (ESR1), encoding amino acids 251–595 (hinge and LBD), was amplified from human breast cDNA by PCR using appropriate primers, which introduced *Eco*RI and *Bam*HI restriction sites, respectively. The PCR product was cloned into *Eco*RI/*Bam*HI digested vector pM (Takara Bio Europe, Saint-Germain-en-Laye, France). The resulting plasmid (pM-ESR1(251–595) encodes a fusion protein of N-terminal GAL4-DBD and the aforementioned region of ERα. Parts of the open reading frame of human AR (NM_000044), encoding amino acids 1–503 (N-terminal region with AF1) and 640–920 (part of hinge and LBD), were amplified by PCR from human testis cDNA and cloned into pVP16­(AD) (Takara Bio Europe) and pM, respectively. The resulting plasmids pVP16-AR (1–503) and pM-AR (640–920) encode fusion proteins of N-terminal VP16 activation domain and GAL4-DBD and the respective regions of AR. The pGL4-G5 firefly luciferase reporter gene plasmid was described previously.^73^ The Renilla luciferase expression plasmid pGL4.75 [hRluc/CMV] was purchased from Promega (Madison, WI, USA).

### Cell Viability

HEK293 cells were seeded at 40,000 cells per well into 96-well plates. On the following day, cells were treated with 10, 30, or 100 μM chemicals for 24 h. Cell viabilities were determined as described previously using the CellTiter-Glo 2.0 assay (Promega).^73^ Each of the three independent experiments was performed in technical triplicates.

### Mammalian One-Hybrid and Two-Hybrid Assays

Per well of a 96-well plate, a plasmid DNA mixture consisting of 0.26 μg of pGL4-G5, 0.04 μg of pM-ESR1 (251–495), and 0.005 μg of pGL4.75 [hRluc/CMV] was diluted with 150 mM NaCl to a final volume of 25 μL. In the case of the N–C-terminal interaction assay of AR, the ESR1 effector plasmid was replaced by 0.02 μg each of pVP16-AR (1–503) and pM-AR (640–920). Aliquot (0.6 μL) of 1 mg/mL PEI MAX 40K solution was diluted to 25 μL with 150 mM NaCl. The diluted PEI MAX 40K was added to the diluted DNA mixture, and the mixture was incubated at room temperature for 15 min. In parallel, HEK 293 cells were adjusted to 40,000 cells in 200 μL of culture medium per well. The transfection mixture (50 μL) was added to the cell suspension, gently mixed, and pipetted into a well of a 96-well plate. The next day, the transfected cells were treated with chemicals for 24 h before cell lysis with 50 μL per well of passive lysis buffer (Promega). Firefly and Renilla luciferase activities were measured as described previously.^73^ Results were normalized for transfection efficiency by dividing firefly luciferase activity by Renilla luciferase activity, measured from the same well. Assays were performed in technical triplicate in each independent experiment. The number of independent experiments (i.e., biological replicates) is indicated in the respective figure legends. Concentration response curves were fitted with nonlinear regression using the formula with a variable slope (four parameters) or the formula with a standard slope (three parameters) of GraphPad Prism 10.4.1 (GraphPad Software, Boston, MA, USA), as indicated in the respective figure legends.

## Results

### Molecular Docking Calculations

The calculated binding affinities of the molecular docking calculations for the EDCs considered in both receptors are presented in Table S1. The results showed that the different compounds do not present any great variations in the binding to AR and ERα (Figure S2A). The native ligands for both AR and ERα (testosterone and 17β-estradiol, respectively) have similar structural features and consequently present similar binding patterns. Furthermore, the structural characteristics of the two receptors (Figure S2B) suggest that the studied EDCs will bind with a pattern similar to those of both receptors and potentially present similar scores. This observation is validated by the similarity in the reported docking scores (Table S1 and Figure S2A). The largest difference between AR and ERα docking scores is observed only for diisononyl phthalate, Hexamoll DINCH, and zearalenone (ΔΔ*G* −1.1, −1.1, and 1.4 kcal mol^–1^, respectively). Zearalenone appears to bind more tightly to ERα than AR, with an ΔΔ*G* of 1.4 kcal mol^–1^ (Figure S2A, red). The trend is reversed for diisononyl phthalate and Hexamoll DINCH that seem to bind tighter to AR than ERα (Figure S2, blue) with the same ΔΔ*G* of −1.1 kcal mol^–1^. Out of the nine EDCs employed for the docking calculations, diethyl phthalate appears to have the least favorable docking score for AR and ERα with docking scores −6.2 and −5.8 kcal mol^–1^, respectively.

Figure 2 presents the best docking conformations of the selected EDCs in the binding cavities of AR and ERα. As a reference, in both cases, we selected the crystal structures of the receptor in complex with the natural substrate (dihydrotestosterone) for the AR^18^ and the known inhibitor drug tamoxifen for ERα.^17^ Bisphenols (Figure 2A), phthalates (Figure 2B), and PFAS (Figure 2C) are docked in a position like the respective crystal structure receptor substrate. Only zearalenone appears to bind in different positions in the AR (Figure 2D, left panel) and ERα (Figure 2D, right panel). In ERα, zearalenone binds away from the site of known inhibitor tamoxifen. This deviation may be explained by the different structure of the compound that involves a large, uncommon ring structure (Table 1). Moreover, these differences in the binding locations predicted by docking may explain the difference observed in the respective docking scores (Figure S2A) between the two receptors. The validity of the docking protocol followed in this paper is further supported by the redocking of the crystal ligands. The protocol, employed, duplicates the crystal conformations in both receptors (Figure S2C,D) with RMSD values 1.6 Å for tamoxifen in ERα and 0.5 Å for dihydrotestosterone in AR compared to their experimentally derived position.

**Figure 2:**
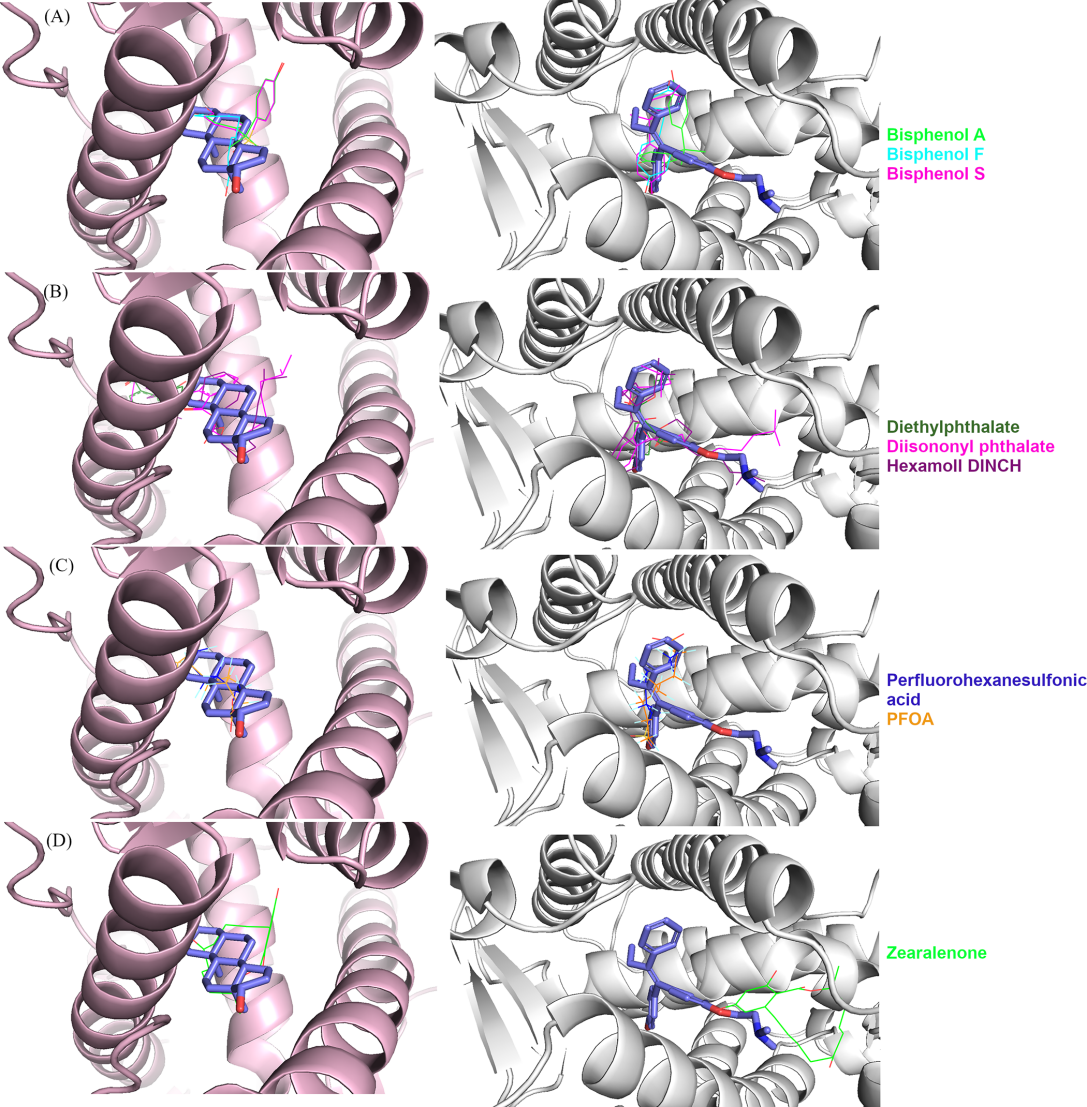
Best scoring conformations, derived from molecular docking calculations, of the: (A) bisphenols, (B) phthalates, (C) PFAS, and (D) mycotoxin zearalenone, in complex with AR (left panel, pink) and ERα (right panel, white). All structures have been superimposed with the crystal structure of the AR in complex with dihydrotestosterone (PDB ID: 1t7t) and the ERα in complex with the inhibitor drug tamoxifen (PDB ID: 3ert). The crystal substrates are depicted as purple stick models, while all docked compounds are depicted as wireframes.

### Experimental Results

Interestingly, these binding differences translated also into experimentally determined differences in potency (EC_50_) and efficacy (*E*_max_) among bisphenols and between bisphenols and zearalenone in the activation of ERα (Figure 3). Zearalenone exhibited a potency that was 120-, 430-, and 1060-fold higher than the potencies of BPA, BPF, and BPS, respectively (Table 2). It also demonstrated higher efficacy than the bisphenols. Among the bisphenols, BPA showed higher potency than BPF and BPS, overall aligning with the relative differences in docking scores for ERα. Within the relevant concentration intervals, no apparent cytotoxicity was observed (Figure S3).

**Figure 3:**
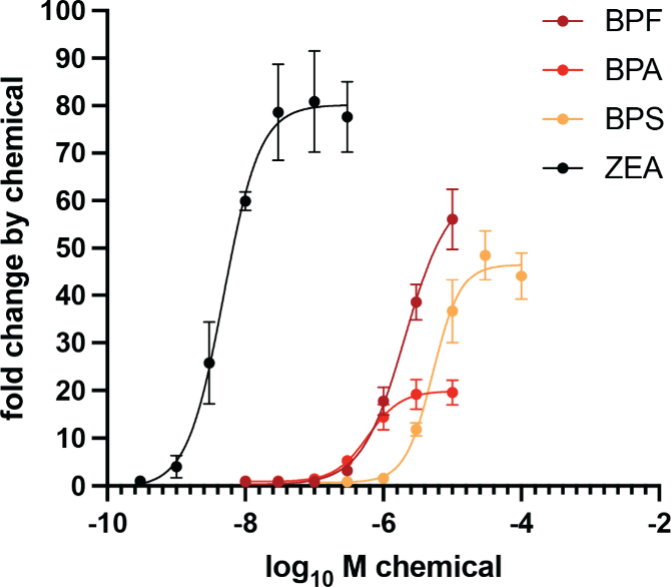
Concentration response curves of ERα activation by bisphenols and zearalenone. ERα mammalian one-hybrid assays were executed in transiently transfected HEK293 cells, which were treated afterward for 24 h with the indicated concentrations of chemicals. Data are presented as a mean ± SD fold change (*n* ≥ 3 independent experiments) with respect to the normalized reporter gene activity of cells treated with 0.1% DMSO only. Curves were fitted with nonlinear regression using the formula with a variable slope (four parameters) of GraphPad Prism version 10.4.1.

**Table 2:** Potency and Efficacy of Bisphenols and Zearalenone in Activating ERα

	EC50 [μM]			
EDC	mean	95% C.I.	*E*_max_ [FC]^a^	conc. [μM]
BPA	0.60	0.46–0.76	19.6	10
BPF	2.1	1.7–2.9	56.1	10
BPS	5.2	4.2–6.5	48.5	30
ZEA	0.0049	0.0036–0.0065	89.9	0.1

a FC, fold change.

Bisphenols are also known to bind to AR and recognized as AR antagonists.^74^ Zearalenone also was shown to exert antagonist activity on AR.^75^ To analyze whether molecular docking scores predict the potency and efficacy of bisphenols and zearalenone as AR antagonists, we made use of AR ligand-dependent dimerization, which relies on the interaction between the LBD and the N-terminal region of AR.^76^ A respective mammalian two-hybrid assay shows that 11-ketoDHT induced the interaction of both regions of AR with an EC_50_ of 3.3 nM (95% CI 2.3–4.7 nM) (Figure 4A). BPA, BPF, and zearalenone antagonized AR dimerization with similar potency and efficacy, while BPS was less potent and efficacious (Figure 4B and Table 3).

**Figure 4:**
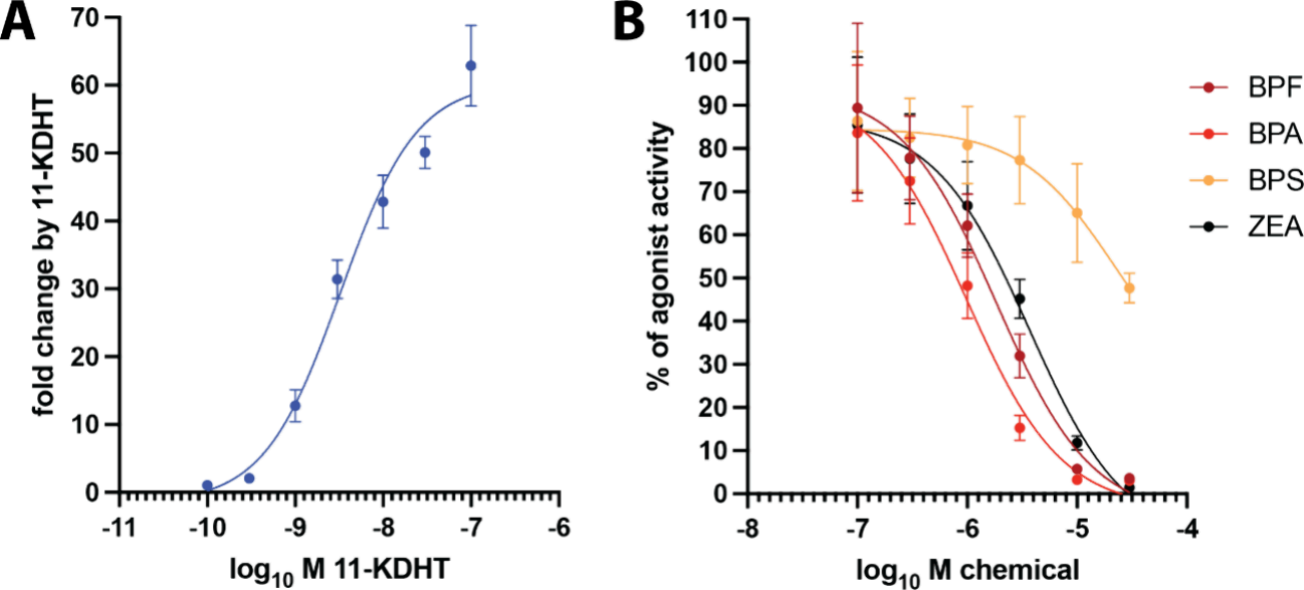
Bisphenols and zearalenone antagonize AR activation. AR N–C-terminal interaction mammalian two-hybrid assays were run in transiently transfected HEK293 cells, which were treated afterward for 24 h with the indicated concentrations of chemicals. (A) Concentration response curve of agonist 11-ketoDHT. Data are presented as a mean ± SD fold change (*n* = 4 independent experiments) with respect to the normalized reporter gene activity of cells treated with 0.1% DMSO only. (B) Inhibition of agonist-induced AR dimerization by concurrent treatment with the indicated concentrations of bisphenols and zearalenone. Data are presented as mean ± SD (*n* = 3 independent experiments) with respect to the normalized reporter gene activity of cells treated with 3 nM 11-ketoDHT only, which was designated as 100%. Curves were fitted with nonlinear regression using the formula with a standard slope (three parameters) of GraphPad Prism 10.4.1.

**Table 3:** Potency and Efficacy of Bisphenols and Zearalenone in Inhibiting AR^a^

	IC50 [μM]			
EDC	mean	95% C.I.	% residual agonist activity	conc. [μM]
BPA	0.98	0.55–1.7	3.1	30
BPF	1.8	1.0–3.3	3.6	30
BPS	23.0	2.9–???	47.6	30
ZEA	3.7	2.0–6.8	1.5	30

a ???, not computable by GraphPad Prism.

### Molecular Dynamics Simulation Analysis

The docked conformations of the compounds served as the initial structures for a series of 18 protein–ligand MD systems (9 per receptor), each run in triplicate (54 trajectories in total). The RMSD analysis of the backbone atoms of the two receptors (Figures S4 and S5) highlights the deviation of the protein structure from its initial conformation. The ERα behavior in the complexes with the various EDCs (Figure S4) shows the receptor structure to be more mobile compared to AR during the simulations (Figure S5). ERα presents higher RMSD values compared to its initial conformation, reaching up to almost 4 Å in the complex with Hexamoll DINCH (Figure S4, middle panel, right). This variation in the RMSD values could be attributed to the loop regions of the ERα that show increased RMSF values (Figure 5A and Figure S6). On the other hand, AR appears less mobile during the MD simulations, with RMSD values not exceeding 2 Å (Figure S5). The highest RMSD values for AR are observed in the complex with perfluorohexanesulfonic acid (PFHxS, Figure S5, bottom panel left), with a mean value of 1.5 Å (±0.17). The most stable conformation for ERα is observed in its complex with perfluorooctanoic acid (PFOA, Figure S4, bottom panel, middle), while in the complex with zearalenone (Figure S4, bottom panel, right), the receptor appears to be more flexible throughout the simulation. The flexibility of the ERα is further supported by the RMSFs (Figure 5A and Figure S6). The backbone protein atoms in ERα appear to be slightly more flexible compared to the values observed for the AR (Figure 5B and Figure S7). In all AR complexes, the residues show small fluctuations from their initial conformation with the highest deviation observed in the area between residues 55–65 and the area 145–155 that reach values of almost 2 Å. This difference in flexibility between the two receptors may indicate that EDCs affect them differently, leading to variations in their interactions. The most important observation is that the receptor areas that are reported in the literature to interact with native ligands and drugs^17,18^ present low RMSF values. This observation points to a potential common binding mechanism for the EDCs studied here.

**Figure 5:**
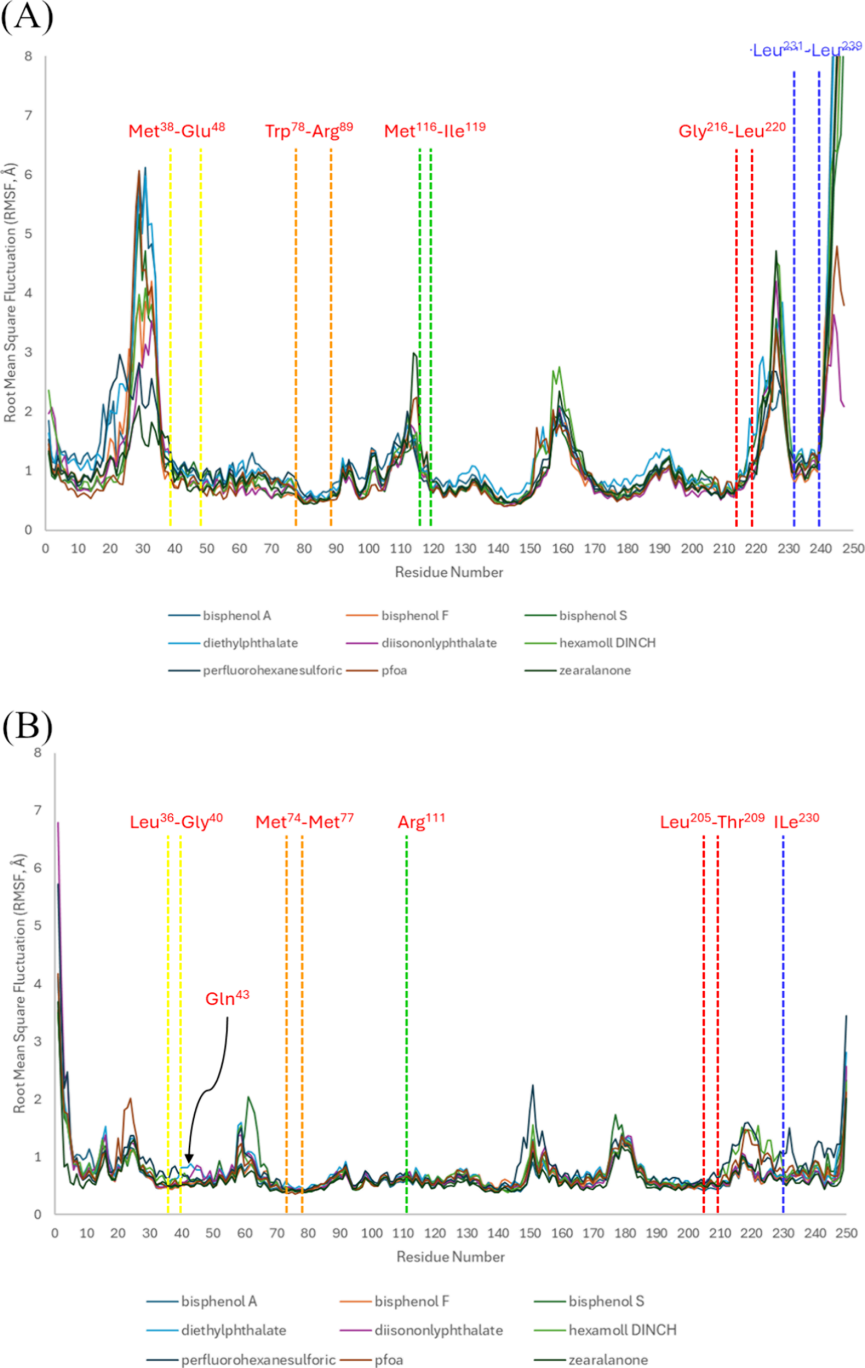
Atomic fluctuations of the receptor residues in the different complexes for (A) ERα, vertical dashed lines indicate boundaries of key secondary-structure elements: Met^38^–Glu^48^ (yellow), Trp^7^ ^8^–Arg^89^ (orange), Met^116^–Ile^119^ (green), Gly^216^–Leu^220^ (red), and Leu^231^–Leu^239^ (blue). (B) AR, vertical dashed lines indicate boundaries of key secondary-structure elements: Leu^36^–Gly^40^ (yellow), Met^74^–Met^77^ (orange), Arg^111^ (green), Leu^205^–Thr^209^ (red), and Ile^230^ (blue). With red letters, the residues were reported to interact with native ligands and drugs in the literature. The RMSF was calculated on Cα atoms relative to the initial docked conformation structure.

The next step in the analysis involved the identification of potential hydrogen bond interactions (HBs) that are observed during the MD simulations. As expected, the hydrogen bond interactions do not vary greatly among the different EDCs. Based on the structures of the compounds (Table 1), there are few groups that can create hydrogen bonds with the residues of the two receptors. The results of the analysis are presented in Tables S2 and S3 and Figure 6. In both receptors, the EDCs studied established ΗΒ interactions with approximately the same residues. In the case of ERα, residues Glu^48^ and His^219^ take part in most of the ΗΒ interactions (Table S2 and Figure 6A). Both residues as well as the others involved in HBs are reported to take part in ligand binding. Consequently, these residues are located in regions of the protein that have low mobility (Figure 5A and Figure S6). Zearalanone binds to an alternative location, distinct from the other EDCs (Figure 2D) and thus creates hydrogen bonds with Thr^42^, Met^223^, and Asn^227^. In the complexes of the various EDCs with the AR, a similar pattern is observed. Most of the HBs formed involve residues Asn^37^ and Gln^43^ (Table S3 and Figure 6B). Although these two residues are not reported to directly participate in the binding process of dihydrotestosterone,^18^ they are located on a helix that interacts with the native ligand (Figure 6B) and keeps it in position inside the binding site. The analysis of the HBs formed between the receptor and the various EDCs further underscores that the residues participating in these interactions are located in regions with low RMSF values (Figure 5 and Figures S6 and S7). The only EDC that does not form any HB interactions, in both receptors, is Hexamoll DINCH. The lack of hydrogen bonding can be attributed to its three-dimensional structure that may not assist in the correct positioning of its oxygen atoms. Out of all of the EDCs studied, diethyl and diisononyl phthalates create strong hydrogen bond interactions in AR (Table S3). In ERα, diethyl phthalate participates in HB with His^219^ (Table S2) that is present for only 10% of the simulation time, while diisononyl phthalate does not create any HB interactions (Table S2).

**Figure 6:**
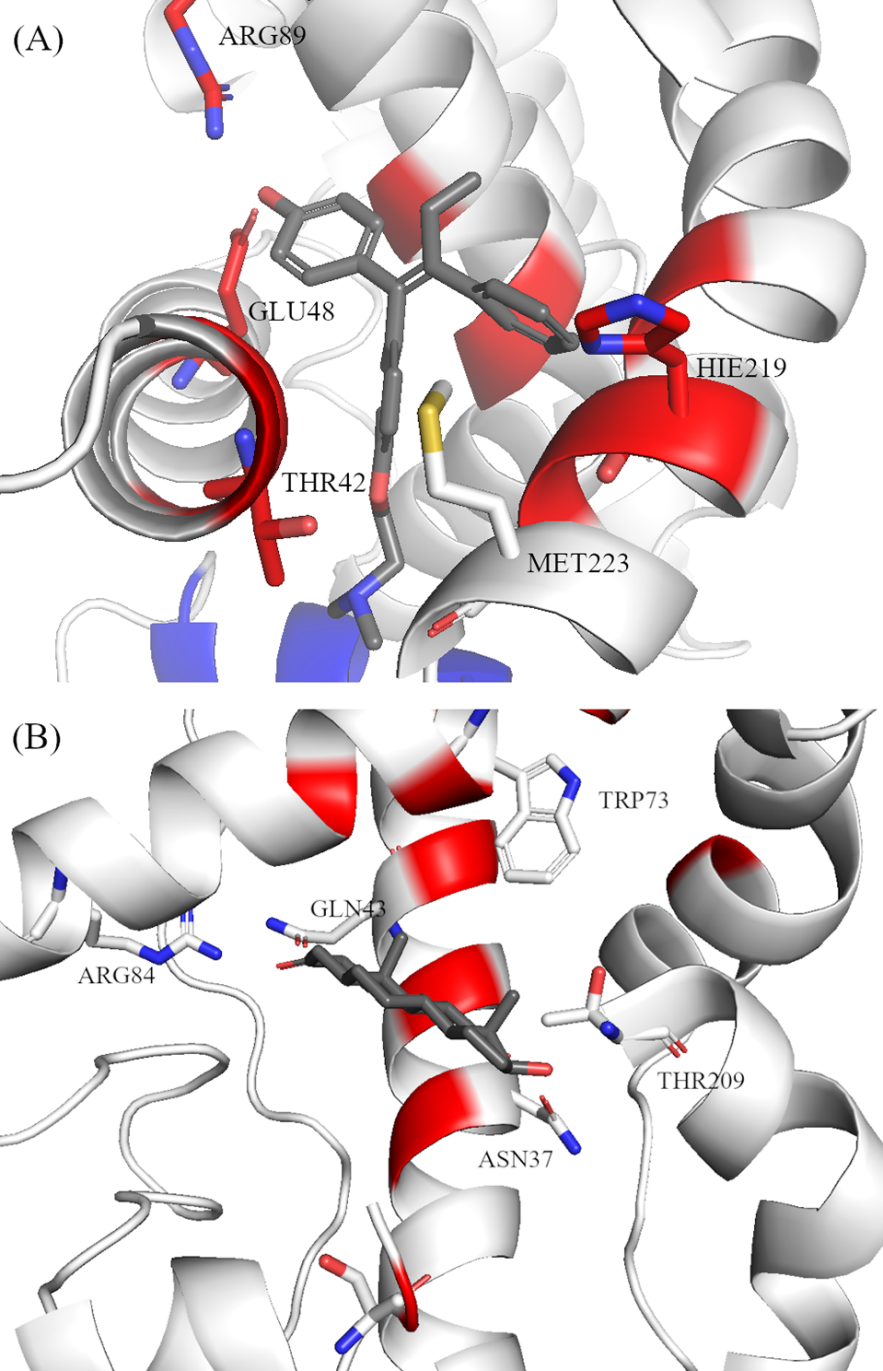
Receptor residues involved in hydrogen bond interactions with the various EDCs over the duration of the MD simulations in ERα (A) and AR (B). In dark gray are the crystal structures of tamoxifen (PDB ID: 3ert) and dihydrotestosterone (PDB ID: 1t7t). Residues highlighted in red are reported to interact with the ligands in the receptors, while in blue is helix 12 of ERα that plays a role in drug binding.^17,18^

A comparison between the crystal structures of ERα complexes with bisphenol A, estradiol, and tamoxifen and the representative conformation derived from MD simulations (Figure 7) reveals a good agreement between the simulated results and those observed in crystallographic studies. Specifically, superposition of the different structures shows that bisphenol A binds to the same region as estradiol (the receptor’s native ligand, Figure 7, gray) and tamoxifen (Figure 6A, gray). As previously reported by Delfosse et al.,^31^ the residues that interact with bisphenol A are mainly His^219^ and Glu^48^ that are also implicated in interactions with estradiol. Moreover, the specific residue side chains have the same orientation as those reported in the crystallographic structures (Figure 7, magenta). It is important to note that the structures solved by Delfosse et al.^31^ contained the mutation Tyr232Ser. The only small discrepancy is observed with regard to Met^116^, which is oriented differently in the simulations compared to the crystal structure (Figure 7, magenta and lime green).

**Figure 7:**
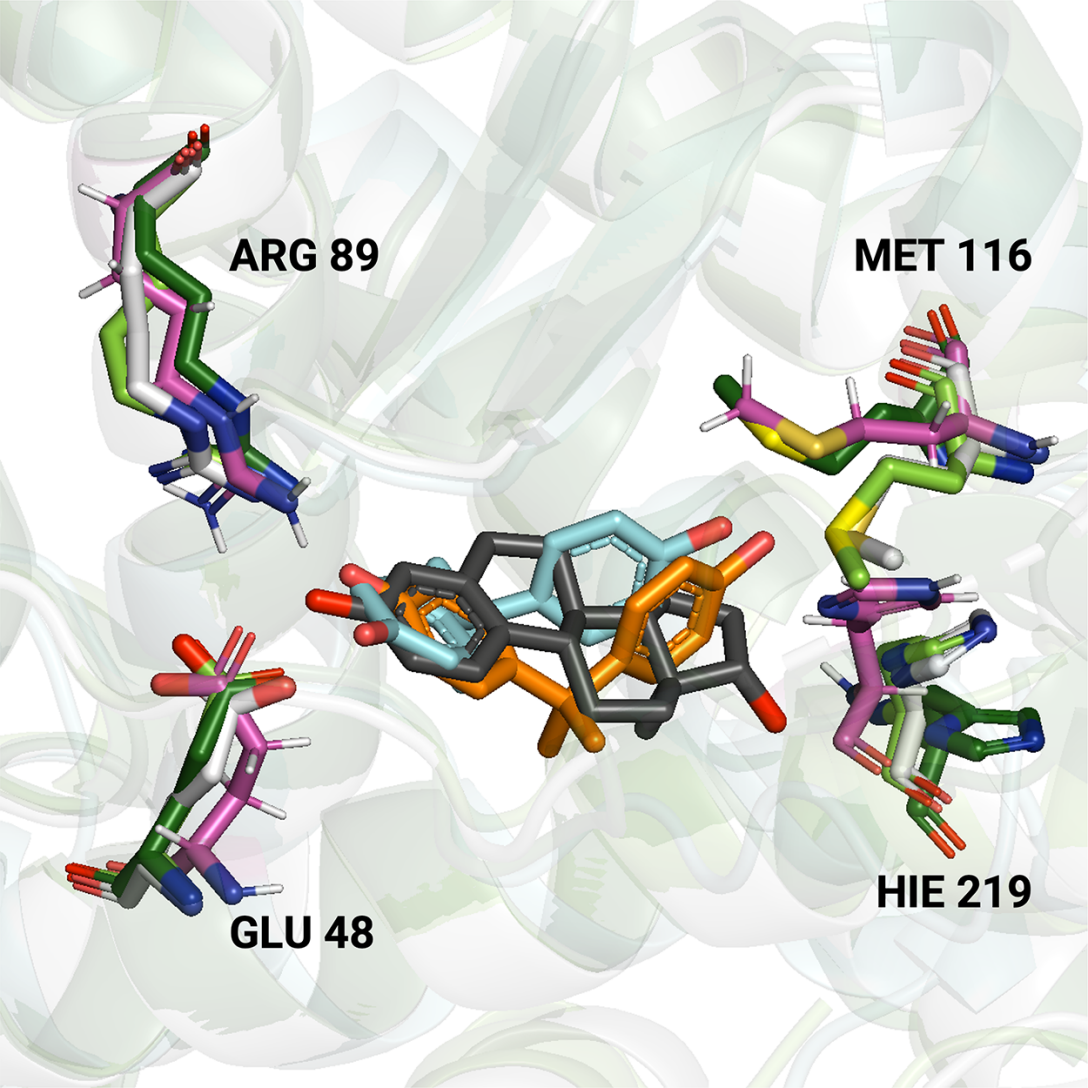
Superimposition of the crystal structures for the complexes of estradiol (PDB ID: 1gwr, gray),^78^ bisphenol A (PDB ID: 3uu7, orange),^31^ and tamoxifen (PDB ID: 3ert)^17^ with the representative conformation of bisphenol A (cyan), derived from the MD simulations. Receptor residues reported to be involved in a hydrogen bond with bisphenol A^31^ are colored lime green. The same residues are colored white in the ERα-estradiol crystal structure, magenta in the MD ERα-bisphenol A complex, and dark green in the ERα-tamoxifen crystal structure.

We compared our findings with those of other computational studies to gain further insights into EDC interactions. As expected, most studies focus on a range of representative EDCs, often exhibiting structural diversity compared with the native ligands or drugs targeting the receptors. This is evident in the PFAS group, where the compounds have linear chains and no ring scaffolds (Table 1). Another distinctive feature of PFOA and PFHxS is the presence of the −COO^–^ and −SO_3_^–^ moieties that are polar, in contrast to the neutral nature of estradiol and testosterone, the endogenous ligands of ERα and AR. Based on the study by Cao et al.,^26^ PFOA was reported to create a hydrogen bond interaction with Arg^89^ of ERα as well as Glu^48^, while His^219^ is considered important in positioning the ligands in the binding cavity. This interaction with Arg^89^ is also reported to be important for PFOA recognition.^32,77^ Our calculations highlight this pattern, since PFOA is found to interact with His^219^, while the polar interaction between ligand and Arg^89^ is substituted by the ligand–Lys^226^ interaction. Similarly, to our knowledge, there are no *in silico* studies that investigate the interactions between the specific phthalates and the two receptors. Nonetheless, in the study by Dahbi et al.,^28^ it was reported that phthalate derivatives create interactions with residues Arg^84^ and Gln^43^ in AR, while in ERα, interactions are formed with residues like Glu^48^. These observations are in agreement with our findings that when phthalates bind to AR, they interact mainly with Arg^84^, while in the estrogen receptor, the phthalates studied seem to interact mainly with His^219^.

### MM-GBSA Analysis

To further understand the binding process of the various EDCs, MM-GBSA calculations were performed on the MD trajectories for all of the complexes. The results are summarized in Figure 8 and Tables S4 and S5. In addition to the calculations of the enthalpic and entropic contributions, decomposition analysis was performed for all complexes (Figures S10–S19). The analysis of the results does not reveal a common pattern between the binding of EDCs to the two receptors. Hexamoll DINCH shows the highest binding energy for both ERα and AR (−30.95 and −36.88 kcal mol^–1^, respectively). This tight binding is enthalpically driven (Figure 8), especially in the AR. The hydrophobic nature of Hexamoll DINCH may further enhance the hydrophobic interactions inside the binding cavities of the receptors; this is mirrored in the highly favorable van der Waals contributions (Tables S4 and S5). Thus, this provides a potential explanation for the tight binding despite the absence of hydrogen bonding interactions. A common trend for most of the EDCs is the tighter binding to AR compared to ERα, with the exception of diisononyl phthalate and diethylphthalate (Figure 8, orange).

**Figure 8:**
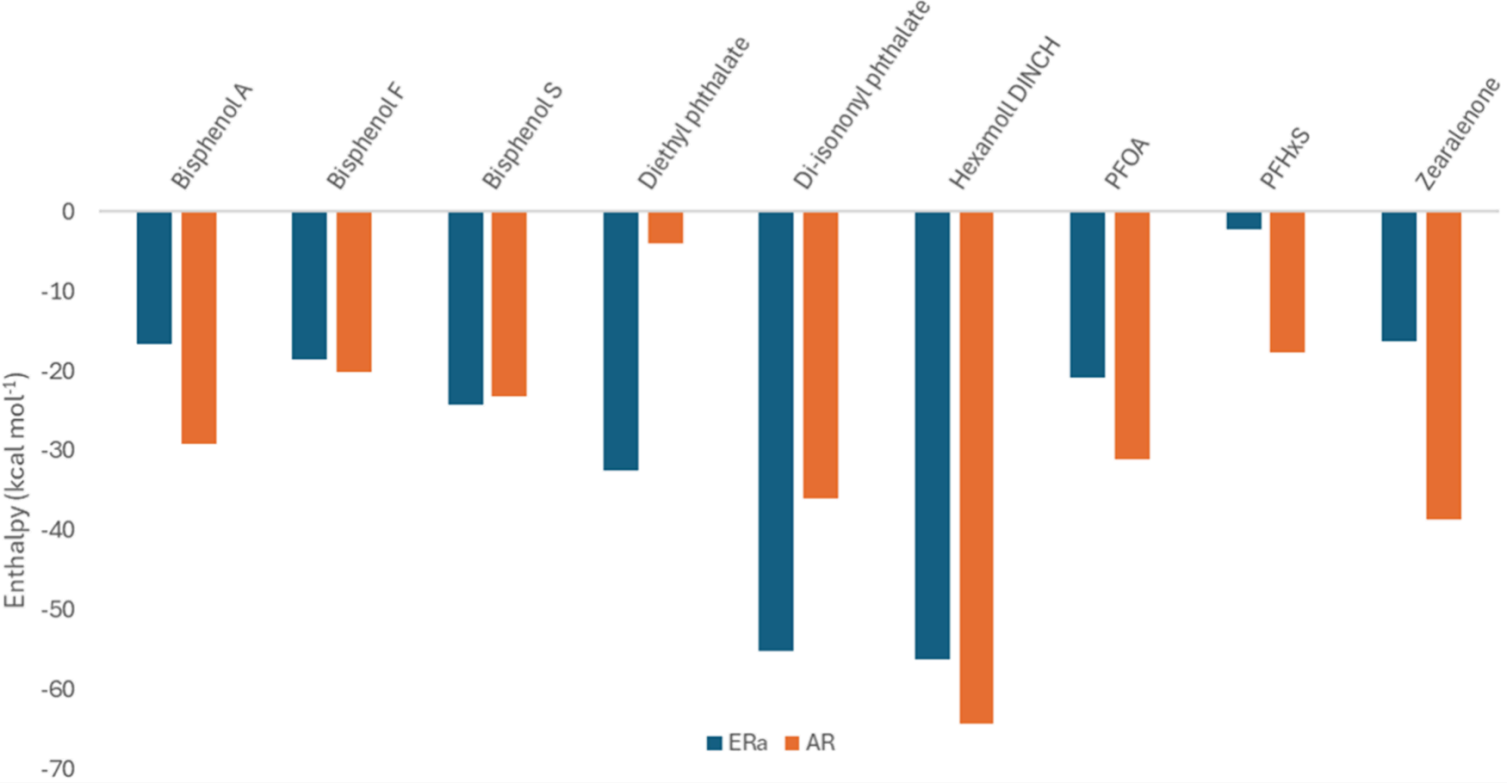
Enthalpic (Δ*H*) contribution to the binding energy (Δ*G*) for the selected EDCs in ERα (blue) and AR (orange). Detailed values, along with the standard error of the mean, for all contributions are presented in Tables S4 and S5.

Another trend derived from the MM-GBSA results is the correlation between the binding affinity and EDC size. Compounds like phthalates (Table 1), which contain a ring scaffold and flexible carbon chains, show increased binding energy for both receptors (Figure 8). On the other hand, compounds like bisphenols, PFOA, and PFHxS display less favorable binding energies. This could possibly be attributed to the fact that we employed only a single molecular entity in our study. Thus, the smaller molecules may show an increased flexibility inside the binding cavity of the receptor. In fact, it has been recently reported that small molecules, such as PFOA, could simultaneously bind to the receptor with two molecules, and these molecules could interact with different sites in the protein’s ligand binding pocket (LBP).^79^ As expected, enthalpy contributions are driving the binding process in all complexes, highlighting the importance of the van der Waals for all the EDCs. For PFOA and PFHxS (Tables S4 and S5), this observation is in agreement with Cao et al.^26^ that have performed similar calculations in the estrogen receptor and PFOA complexes and have reported that the binding energy is lower than the one reported for native ligands.

### Alchemical Relative Binding Free Energy Calculation Analysis

To compute the relative binding free energies of bisphenol F to ERα and AR—compared to bisphenols S and A—MD simulations were combined with relative free energy calculations using a nonequilibrium approach to accelerate convergence. Initially, a 10 ns production run in the NPT ensemble was performed for each ligand pair. To assess the convergence of the calculations, the three ligands were connected through a closed thermodynamic cycle, allowing the evaluation of hysteresis. In a well-converged alchemical free energy calculation, the sum of the free energy changes around the closed cycle should be zero or near zero. For the AR–ligand complexes, the cycle closure error was 0.68 ± 2.25 kcal mol^–1^, while for the ERα–ligand systems, it was −0.37 ± 3.20 kcal mol^–1^. These values indicate that the total deviations were not minimal, as both cycles showed high standard deviations. To address this, we extended the equilibrium NPT calculations for the bisphenol S → bisphenol F perturbation, which had exhibited the highest standard deviation among replicas (2.74 kcal mol^–1^ for ERα and 2.01 kcal mol^–1^ for AR) from 10 to 20 ns. The additional sampling resulted in a substantial reduction in the standard deviation for this perturbation (0.62 kcal mol^–1^ for ERα and 0.60 kcal mol^–1^ for AR). Consequently, the standard deviation of the cycle closure errors also decreased markedly, yielding 0.05 ± 1.58 kcal mol^–1^ for ERα and −0.48 ± 1.43 kcal mol^–1^ for AR, demonstrating improved convergence relative to the original simulations. The calculated energies (Table 4) indicate that bisphenol A presents an improved binding affinity compared to bisphenol S and similar to F to both ERα and AR, with an approximate 4 kcal mol^–1^ preference for ERα over AR. Specifically, the relative binding free energy (ΔΔ*G*) of bisphenol S compared to bisphenol A was 4.38 ± 0.91 kcal mol^–1^ for AR and 7.46 ± 1.38 kcal mol^–1^ for ERα. In addition, bisphenol F displayed stronger binding than bisphenol S, with ΔΔ*G* values of −3.83 ± 0.60 kcal mol^–1^ for AR and −7.07 ± 0.62 kcal mol^–1^ for ERα. Finally, the RBFE calculations comparing the binding of bisphenol A and bisphenol F to AR and ERα indicate similar binding affinities for both proteins. The relative binding free energies (ΔΔ*G*) of bisphenol F compared to bisphenol A were 1.03 ± 0.92 kcal mol^–1^ for AR and 0.34 ± 0.44 kcal mol^–1^ for ERα, suggesting that these two ligands exhibit comparable affinities toward both targets.

**Table 4:** Binding Free Energy Differences in (kcal mol^–1^) for Each Perturbation and the Reported Cycle Closure Error

	ΔΔ*G*	
perturbation	ERα	AR
bisphenol A → bisphenol F	0.34 ± 0.44	1.03 ± 0.92
bisphenol S → bisphenol F	–7.07 ± 0.62	–3.83 ± 0.60
bisphenol A → bisphenol S	7.46 ± 1.38	4.38 ± 0.91
cycle closure error	0.05 ± 1.58	–0.48 ± 1.43

To assess the free energy convergence of the calculations, the work values were obtained from the nonequilibrium thermodynamic integration (TI) simulations, along with the corresponding work distributions for the ERα and AR complexes (Figures S20–S22 and S23–S25, respectively). The work distributions show a strong overlap for the solvation part (Figures S20–S25, left panels) and an adequate overlap for the complex part (Figures S20–S25, right panels) of the thermodynamic cycle across all systems. The observed overlap between forward and reverse work distributions is a robust indicator of sampling quality and convergence in simulations of free energy. The results from the alchemical relative binding free energy calculations analysis are in relative agreement with the MM-GBSA calculations (Figure 8) that show bisphenols F and S to have similar enthalpy for both receptors, while bisphenol A appears to have a stronger affinity for AR. These results are consistent with experimental assays, which show that bisphenol A and bisphenol F bind more strongly to AR and ERα than bisphenol S.

## Conclusions

The scope of this study was to employ computational and experimental methods for the investigation of the binding patterns of EDCs in hormone receptors such as ERα and AR. The *in silico* analysis showed that there are no significant conformational changes in the receptors upon EDC binding. Moreover, it was not possible to observe any major conformational changes in the movement of helix 12, which is considered to play an important role in ligand binding. This could be attributed to the fact that the receptors are reported to exert their action via the formation of hetero- and homodimers upon ligand binding.^30,80,81^ Therefore, the interplay between the monomers could affect the helix positioning and movement between the monomers.

Regarding estrogen receptor binding, it was observed that in the complexes, the hydrogen bond interactions of the EDCs are mainly formed with Glu^48^ and His^219^ (Figures 7A and 8 as well as Table S2). Both residues are reported to have a role in native ligand binding^82,83^ highlighting a similar binding pattern for EDCs and estrogens. Additionally, the performed simulations showed that interactions arise with Thr^42^ (ERα-zearalenone), Arg^89^ (ERα-PFHxS), Glu^114^, and Met^223^. All of these residues are located in regions surrounding the binding cavity of the receptor. Although our calculations employed the antagonist form (PDB ID: 3ert) of the estrogen receptor, we observed that the interaction patterns for all EDCs studied were consistent with those reported for both the natural substrate (estradiol)^78^ and antagonists such as tamoxifen.^17^ Moreover, our simulations showed a similar pattern of interactions for bisphenols with those reported by Rashidian et al.^83^ Similarly, the interactions in AR follow an analogous pattern, as EDCs were calculated to form hydrogen bonds mainly with Asn^37^, Gln^43^ Arg^84^, Ser^110^, and Thr^209^. Notably, the interactions observed with Arg^84^ and Gln^43^ are closely aligned with those reported in previous studies.^18,45,46,83,84^ Despite EDC chemical diversity, our analysis has identified a common binding pattern for both ERα and AR receptors that closely resembles that of known drugs and native ligands.

Moreover, MM-GBSA analysis showed that van der Waals interactions are the dominant contributions to the binding free energy. This is attributed to the absence of polar groups from most compounds that could facilitate other types of interactions with the receptors. While MM-GBSA may occasionally over- or underestimate absolute binding free energies and can show relatively high standard deviations (>5 kcal mol^–1^), it still accurately captures common EDC binding features. Therefore, the reported calculated values from MM-GBSA calculations can be employed to identify potential binding patterns and trends in the various complexes. Furthermore, the results presented in Tables S4 and S5 highlight the agreement with the reported experimental values. The calculated Δ*G* values for bisphenol A are −7.88 kcal mol^–1^ for ERα and −6.92 and −5.37 kcal mol^–1^ for bisphenols F and S, respectively (Table S4). Similarly, in AR the respective values are −9.77, −8.13, and −3.34 for bisphenols A, F, and S (Table S5), respectively. This trend in the MM-GBSA Δ*G* values mirrors the observed results in the experiments. From the three bisphenols studied, BPA showed the highest potency against ERα (Table 2) and the highest inhibitory activity against AR (Table 3) followed by BPF and BPS. On the other hand, the calculations do not accurately represent the potency of zearalenone. This could be attributed to the uncommon ring scaffold of the compound that could not be accurately modeled throughout the simulation. Accordingly, alchemical relative binding free energy calculations were used to compare compounds sharing a common scaffold with minor structural variations and to determine how the changes in small groups inside the molecules can impact binding affinity. Bisphenols A-S are such compounds, and the relative binding energy analysis indicates that chemical modifications to the compound scaffold can lead to varying effects on binding affinity. Among the studied compounds, bisphenol S shows the weakest binding, consistent with experimental observations (Table 4). However, due to the chemical variability of phthalates and PFAS, it was not possible to employ alchemical relative binding free energy calculations to these compounds. The variation in binding energies observed between the EDCs is also a function of their size and chemical structure.^26^ Moreover, it is important to highlight the possibility that EDCs like PFOA can bind with two molecules simultaneously to the receptor.^79^

In conclusion, despite their chemical diversity, EDCs display consistently favorable binding toward estrogen and androgen receptors through similar interaction patterns, underpinning their biological effects. Compounds, such as PFAS derivatives, show extensive accumulation and a long half-life in the human body. For example, their distribution in blood and liver highlights their potential to cause adverse effects^85,86^ either as antagonists or as a potential partial activator of ERa in the case of PFOA.^27,87^ In this context, molecular dynamics simulations can identify these common features and assist in better understanding the impact of these compounds. Additionally, alchemical relative free binding energy calculations can predict which chemical modifications in a specific scaffold lead to increased activity and, thus, offer a potential evaluation metric for risk assessment. The analysis at the atomistic level could highlight potential molecular initiating events (MIE), such as the inhibition or stimulation of the receptors, that lead to imbalances in hormone homeostasis and hormonal regulation patterns.

## Supplementary Material



## Data Availability

All calculations were performed using the open-source software Autodock Vina^52^ (molecular docking), OpenMM^53^ (molecular dynamics simulations), AMBER Tools^62^ (MM-GBSA analysis), and GROMACS^68^ (Alchemical Relative Binding Free Energy Calculations). All molecular structures, activity data, GROMACS and OpenMM parameter files, and processed outputs are available in the Supporting Information and, upon publication, at Zenodo (doi: 10.5281/zenodo.15738442). Due to commercial licensing restrictions, the Asclepios Enalos KNIME nodes used for workflow construction are proprietary and cannot be publicly shared. The results of the analysis and the respective files are fully disclosed.

## Notes

Detailed information regarding the setup of molecular docking and molecular dynamics simulations: protein and compound structure preparation, molecular docking simulations setup, and parameters; molecular dynamics simulations system setup and parameters; additional parameters for MM-GBSA calculations, parameters for alchemical relative binding free energy calculations; additional results for molecular docking: binding affinities for the complexes and graphical representations of redocked ligands; and additional results for molecular dynamics simulations: RMSD for backbone protein atoms and ligands, hydrogen bond analysis, and per-residue energy decomposition in the various complexes (PDF)
