# Supplementary material for: Assessment of the Binding Patterns for Endocrine Disrupting Chemicals in Complex with Estrogen and Androgen Receptors by Leveraging the Asclepios Enalos KNIME Nodes
Source: J Chem Inf Model. 2025 Oct 20;65(21):11950–64. doi: 10.1021/acs.jcim.5c01437 (PMC12606622; doi:10.1021/acs.jcim.5c01437)
Supplement: Supplementary file 1 [file ci5c01437_si_001.pdf]

Assessment of the binding patterns for endocrine disrupting chemicals in complex with estrogen and androgen receptors by leveraging the Asclepios Enalos KNIME nodes

Haralampos Tzoupis,[1,2] Michail Papadourakis,[3] Konstantinos D. Papavasileiou,[1,2,3] Oliver Burk,[4,5] Volker M. Lauschke,[4,5,6,7] Andreas Tsoumanis,[1,2,3] Georgia Melagraki,[8] Antreas Afantitis[1,2,3,]*

[1]Department of ChemoInformatics, NovaMechanics Ltd., Nicosia CY-1070, Cyprus
[2]Division of Data Driven Innovation, Entelos Institute, Larnaca CY-6059, Cyprus
[3]Department of ChemoInformatics, NovaMechanics MIKE., Piraeus GR-185 45, Greece
[4]Dr Margarete Fischer-Bosch Institute of Clinical Pharmacology, Stuttgart, Germany
[5]University of Tübingen, Tübingen, Germany
[6]Department of Physiology and Pharmacology and Center for Molecular Medicine, Karolinska Institutet and University Hospital, Stockholm, Sweden
[7]Department of Pharmacy, the Second Xiangya Hospital, Central South University, Changsha, China
[8]Division of Physical Sciences and Applications, Hellenic Military Academy, Vari 16672, Greece

*E-mail: afantitis@novamechanics.com

Table of contents

Materials and Methods……………………………………………………………………2
Figure S1…………………………………………………………………………………7
Table S1…………………………………………………………………………………7
Figure S2…………………………………………………………………………………8
Figure S3…………………………………………………………………………………9
Figure S4…………………………………………………………………………………10
Figure S5…………………………………………………………………………………11
Figure S6…………………………………………………………………………………12
Figure S7…………………………………………………………………………………13
Figure S8…………………………………………………………………………………14
Figure S9…………………………………………………………………………………15
Table S2…………………………………………………………………………………15

Table S3……………………………………………………………………..………………16
Figure S10…………………………………………………………...…………………16
Figure S11………………………………………………………………………………...…17
Figure S12………………………………………………………………………………...…17
Figure S13………………………………………………………………………………...…18
Figure S14………………………………………………………………………………...…18
Figure S15………………………………………………………………………………...…19
Figure S16………………………………………………………………………………...…19
Figure S17………………………………………………………………………………...…20
Figure S18………………………………………………………………………………...…20
Figure S19………………………………………………………………………………...…21
Table S4………………..…………………………………………………………………22
Table S5……………………………...………………………………………………………23
Figure S20…………………………………...……………………………………………24
Figure S21……………………………………………………..……………………………25
Figure S22……………………………………………………………………………..……26
Figure S23………………………………………………………………………………...…27
Figure S24……………………………………………………………………………………...28
Figure S25……………………………………………………………………………………...29
References……………………………………………………………………………………...30

Materials and Methods

Protein and compound structure preparation. The crystal structures of: (i) the Estrogen Receptor α in complex with the drug tamoxifen (PDB ID 3ert) [1] and (ii) the Androgen Receptor in complex with 5α-dihydrotestosterone (PDB ID 1t7t) [2] were employed for the molecular docking and molecular dynamics simulations. The preparation of the protein structures was performed with the AsclepiosPDBFixer [3] KNIME node (Figure 1A, main text). The A chain of the receptors was retained, and all heteroatoms were removed, followed by the addition of hydrogen atoms at neutral pH (i.e. pH = 7.4). All protein residues in the files have been renumbered, starting from the first residue.

The structures of the EDCs studied (Table 1, main text), were downloaded from the PubChem database [4]. Open Babel [5] was employed for the conversion of the chemical structures to the SDF format. The hydrogen atoms were added at pH 7.4, using the AsclepiosAddHydrogen node, and the 2D structures were converted into low-energy 3D conformations by implementing the AsclepiosGenerate3Dcoordinates node (Figure 1). All the nodes required for ligand and protein structure preparation are incorporated in the Enalos Asclepios KNIME platform [6–8].

Molecular docking simulations. All simulations were performed using Autodock Vina [9] software as implemented in the Enalos Asclepios KNIME nodes. The compounds and the receptor structures were prepared by using the AutodockFR suite [10]. The Kollman United Atom [11] scheme and the Gasteiger method [12] were employed to add the partial atomic charges for the proteins and the ligands, respectively. The docking boxes for each receptor were centred at the crystallographic ligands (tamoxifen and 5α-dihydrotestosterone) for ERα and AR, respectively, with a grid spacing of 0.375 Å and exhaustiveness level of 8, as implemented in the AsclepiosVina KNIME node. For ERα the coordinates (x, y, z) of the active site were 30.1854, -2.9331 and 23.417 and the box size was set at 21 Å x 24.5 Å x 19.5 Å, while for AR the coordinates were -0.887476, 3.07968 and 36.9992 and the box size was 31 Å x 24 Å x 15 Å.

Molecular dynamics (MD) simulations. The preparation of the protein-EDC complexes was performed by using the workflow depicted in Figure 1A, in the main text. The best scoring docking conformation of each compound was employed as the initial conformation for the MD simulations. All simulations were performed with the OpenMM 7.5 software [3] as part of the Asclepios KNIME workflow. The geometry optimization for all ligands was performed at the B3LYP/6-31G* level [13–15] of theory with the Quantum Mechanics (QM) node of Enalos Asclepios workflow, which utilizes GAMESS-US v2020.2 [16]. The partial atomic charges were derived with the RESP methodology [17]. The AntechamberPlus and the AsclepiosAmberSystemsPrep nodes, implemented in the Enalos Asclepios KNIME nodes, were used for constructing the parameters of each protein-compound complex. The AMBER14SB [18] and the Generalized Amber Force Field (GAFF) [19] were employed for the building of the parameters for the protein and respective ligands. All protein-EDC complexes were solvated in water, using the SPC/E [20,21] model and a truncated octahedron solvent box with a 10 Å buffer distance. Periodic boundary conditions were applied in all directions and the total charge of the systems was neutralized by adding the respective number of Na+/Cl- ions.

The systems were subjected to energy minimization with positional restraints on the proteins and ligand using a harmonic force constant. The restraint force gradually decreased every 5,000 steps

from 100 to 20 to 2 kcal mol^{-1} Å^{-2} until fully abolished. The heating of the system was performed to the target temperature of 300 K for 200 ps, in the canonical ensemble (NVT), using the Langevin thermostat, [22] followed by pressure equilibration at 1 atm in isobaric-isothermal conditions (NPT ensemble). The pressure equilibration was performed using a Monte Carlo barostat [23,24]. Both heating and pressure equilibration were performed by imposing positional restraints on the proteins and ligand atoms with a harmonic force potential of 20 kcal mol^{-1} Å^{-2}. Finally, the production run was performed for 200 ns with no positional restraints imposed on the proteins and ligands in the NPT ensemble. All long-range electrostatic interactions were calculated using the Particle Mesh Ewald method [25] and a cutoff distance of 10 Å was employed for both electrostatic and Lennard-Jones interactions. All bonds involving hydrogen atoms were constrained to their equilibrium distance. The trajectory analysis was performed within the AsclepiosAmberMDSimulation node with the cpptraj module of the AMBER Tools20 software [26]. Root-mean-square deviation (RMSD) analysis involved the backbone atoms of the receptor and all EDCs' heavy atoms, mass-weighted and calculated with respect to the initial system coordinates. Root-mean-squared fluctuations (RMSF) were calculated for the heavy atoms in the protein's backbone, and a mass-weighted average is performed to obtain a single value per amino acid. The hydrogen bond analysis was carried out using geometric criteria for the definition of hydrogen bond interactions, namely, a distance cutoff of 3.5 Å and the angle of donor–hydrogen–acceptor at 150°. The clustering analysis was performed using the dbscan algorithm. For all the analyses of the trajectory files the cpptraj[27] module was employed.

Molecular Mechanics Generalised Born Surface Area analysis (MM-GBSA). The estimation of the relative ligand binding affinities was performed using the MM-GBSA methodology. The interaction energy in the gas phase is calculated using molecular mechanics, while the solvent contribution is estimated using the Generalised Born approximation [28,29]. The method has been shown to perform well in predicting relative binding affinities, despite not always being able to reproduce the experimental results correctly [30]. The calculations for the protein complexes were conducted with the AsclepiosAmberMMPBGBSA node, using the Enalos Asclepios implementation of the AmberTools21

MM-GBSA.py script [26] of the respective KNIME workflow node over the 1,000 last trajectory frames. All figures were prepared using the UCSF Chimera software [31].

Alchemical Relative Binding Free Energy Calculations. A non-equilibrium free energy workflow [32–34] was employed to calculate the relative binding free energies of bisphenol F to ERα and AR, in comparison with bisphenols S and A. Bisphenols S and A were structurally aligned in UCSF Chimera [31] using the dominant cluster of bisphenol F bound to ERα and AR, as obtained from the MD simulations. In the resulting relative binding free energy (RBFE) network (Figure S1), each edge corresponds to both forward and reverse alchemical transformations carried out in both the bound and unbound states. A closed thermodynamic cycle is also formed, enabling the assessment of hysteresis, since the total free energy change around a closed loop should ideally be zero. The parameters for the proteins and the ligands were built using the same method described above. Following ligand parameterization, hybrid structures and topologies for each ligand pair were generated using the pmx [35] module of GROMACS 2024.4 [36]. The atom mappings between the two ligands were created based on a predefined set of rules designed to minimize structural perturbation and maintain simulation stability. Firstly, pmx identifies the maximum common substructure between the molecules to serve as a foundation for the mapping; and secondly, it superimposes the ligands and performs the mapping process based on interatomic distances. The mapping that includes the highest number of directly transformable atoms is selected and the process ensures that no ring structures are fragmented and no disconnected fragments are introduced. The final mapping is then used to generate hybrid structures and topologies using a single topology approach. Simulation systems for both the solvated ligands and the ligand–protein complexes were prepared by placing the molecules in cubic boxes, ensuring a minimum distance of 15 Å between the solute and the box boundaries. Sodium and chloride ions were added to neutralize the overall charge and to achieve a physiological salt concentration of 150 mM.

For each ligand pair, simulations were carried out for both physical end states—state A and state B—corresponding to ligand 1 and ligand 2, respectively. The systems were first subjected to energy minimization, followed by a 500 ps equilibration in the NVT ensemble at 310 K. This was

followed by a 10 ns production run in the NPT ensemble at 310 K and a pressure of 1 bar. A total of 96 snapshots were uniformly extracted from each trajectory, excluding the initial 0.6 ns to allow for additional system equilibration. An exception was made for the Bisphenol S → Bisphenol F perturbation, where a longer 20 ns production run was carried out. In this case, 96 snapshots were also uniformly extracted, excluding the first 1.2 ns to allow for additional equilibration. Alchemical transitions were then initiated from each of these snapshots, in both forward (A to B) and reverse (B to A) directions. Each transition was carried out over a period of 50 ps.

Temperature control in the simulations was achieved using Langevin dynamics [37] with a collision frequency of 1 ps^{-1}. Pressure was maintained at 1 bar using the Parrinello–Rahman barostat [38], with a time constant of 2 ps and a compressibility of 4.5×10^{-5} bar^{-1}. All bonds involving hydrogen atoms were constrained using the LINCS algorithm [39]. Long-range electrostatic interactions were handled using the Particle Mesh Ewald (PME) method [25], with a real-space cutoff of 12 Å, a Fourier grid spacing of 1 Å, and a relative interaction strength at the cutoff set to 10^{-6}. Short range electrostatic and van der Waals interactions were calculated with a cutoff of 12 Å and a switching distance of 10 Å. For the alchemical transitions, non-bonded interactions were managed using a modified soft-core potential to ensure stable transformations. Pmx was used to calculate the free energy estimates from the bidirectional work distributions using a maximum likelihood estimator [40] based on the Crooks fluctuation theorem [41]. Uncertainties were assessed using bootstrap resampling. For each protein–ligand complex, the non-equilibrium workflow was performed in triplicate and the reported binding free energies (ΔGbind average) represent the mean across the replicates. Statistical uncertainties are provided by propagating the errors of the respective calculations.

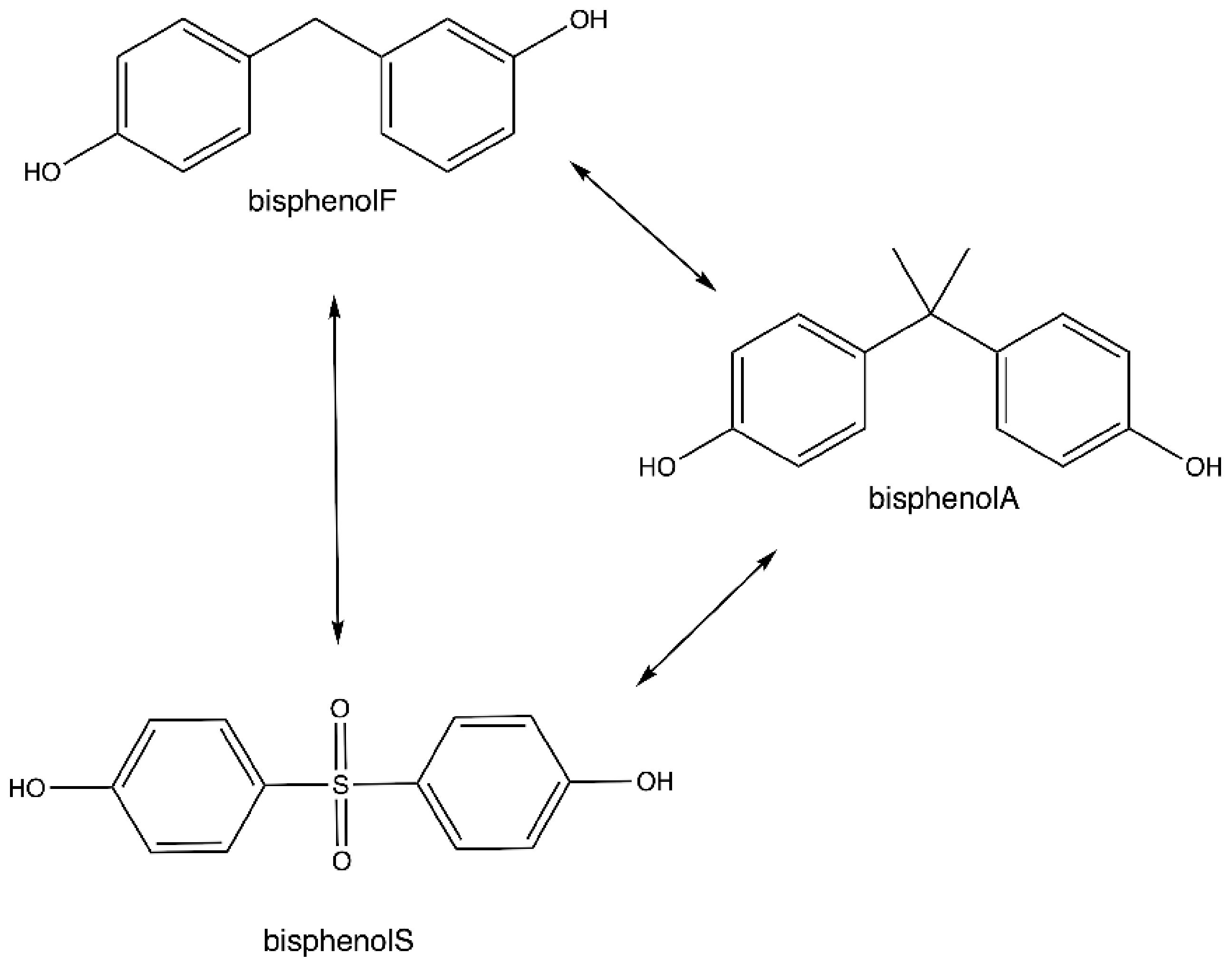

Figure S1. Schematic representation of the relative binding free energy (RBFE) network employed for the alchemical free energy calculations for the three bisphenol compounds.

Table S1. Binding affinities (in kcal mol^{-1}) calculated with Vina-GPU for the EDCs considered.

No.	Name	PubChem ID	Binding affinity		
			AR	ERα	$\Delta\Delta G^a$
1	Bisphenol A	6623	-8.2	-8.2	0.0
2	Bisphenol F	12111	-7.4	-7.3	-0.1
3	Bisphenol S	6626	-7.5	-7.5	0.0
4	Diethyl phthalate	6781	-6.2	-5.8	-0.4
5	Diisononyl phthalate	6787	-7.6	-6.5	-1.1
6	Hexamoll DINCH	11524680	-7.5	-6.4	-1.1
7	PFOA	9554	-8.4	-8.4	0.0
8	PFHxS	67734	-7.9	-8.0	0.1
9	Zearalenone	5281576	-8.1	-9.5	1.4

$^a\Delta\Delta G$= Score AR- Score ERα

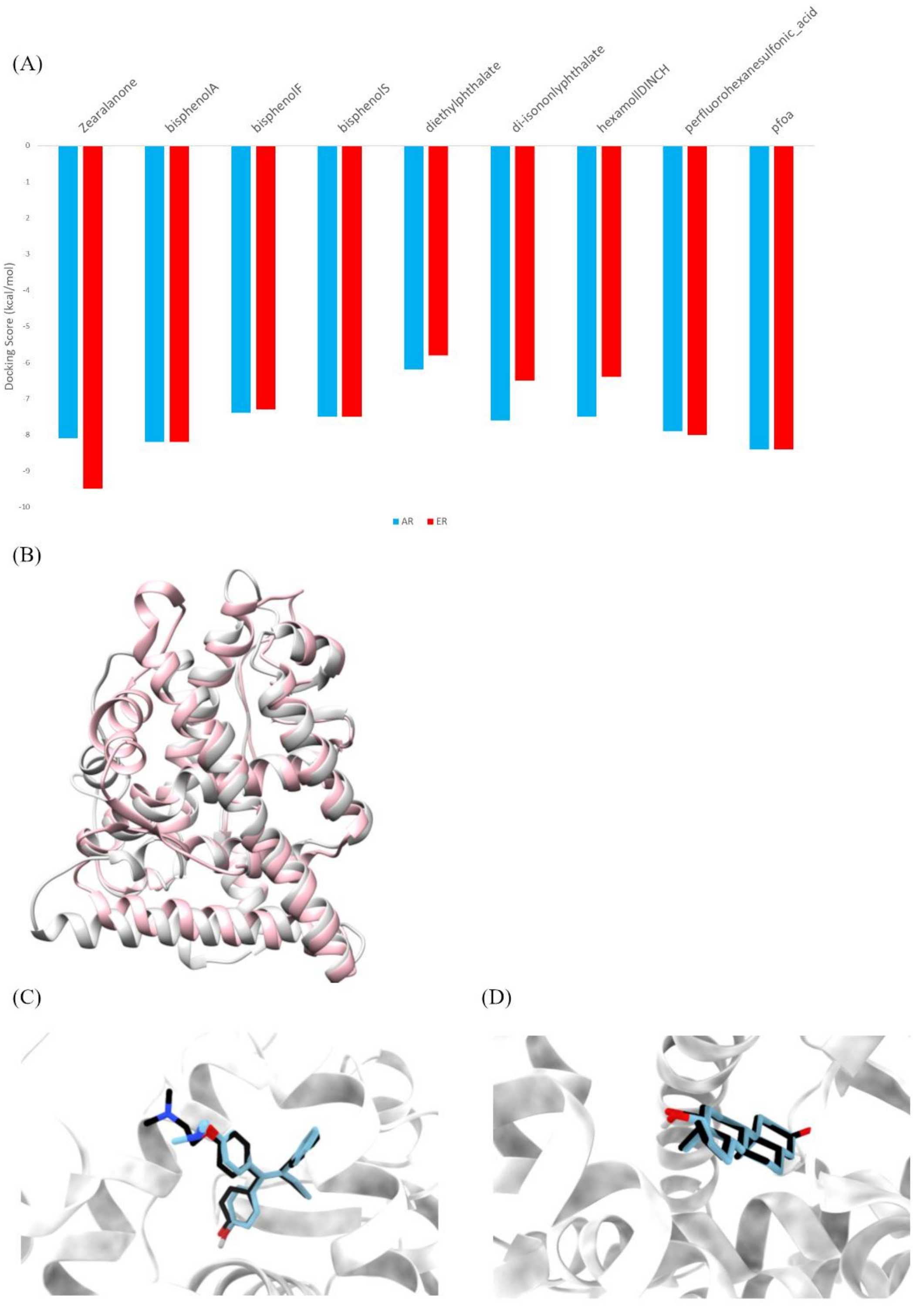

Figure S2. (A) Graphical representation of the docking scores calculated by Autodock Vina, (B) superimposition of the AR (pink) and ERα (light gray) crystal structures (PDB IDs: 1t7t and 3ert respectively), (C) the redocked (cyan) and crystal (black) conformation of tamoxifen in the ERα and (D) the redocked (cyan) and crystal (black) conformation of dihydrotestosterone in the AR.

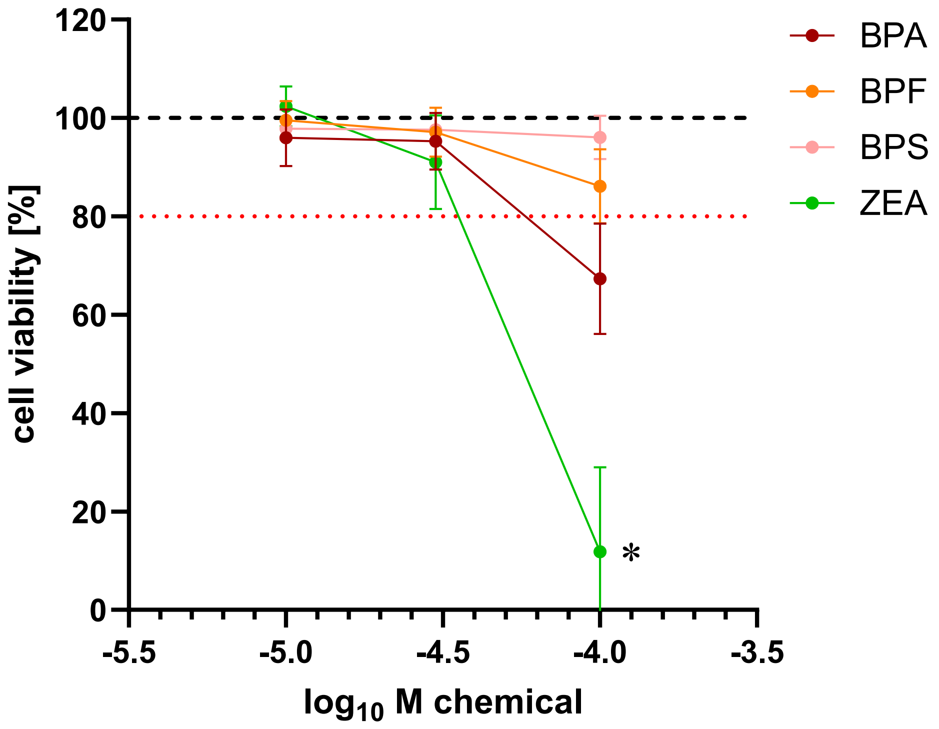

Figure S3. Toxicity of bisphenols and zearalenone in HEK293 cells. Viability was analyzed following 24 h treatment and compared to the viability of cells treated with vehicle (0.1% DMSO) at the same time point, which was set as 100%. Results are presented as mean ± S.D. (n=3 independent experiments). Statistical significant differences to 100% viability were analyzed by one-sample *t*-test, with *P*-values adjusted by the method of Bonferroni. * $P<0.05$.

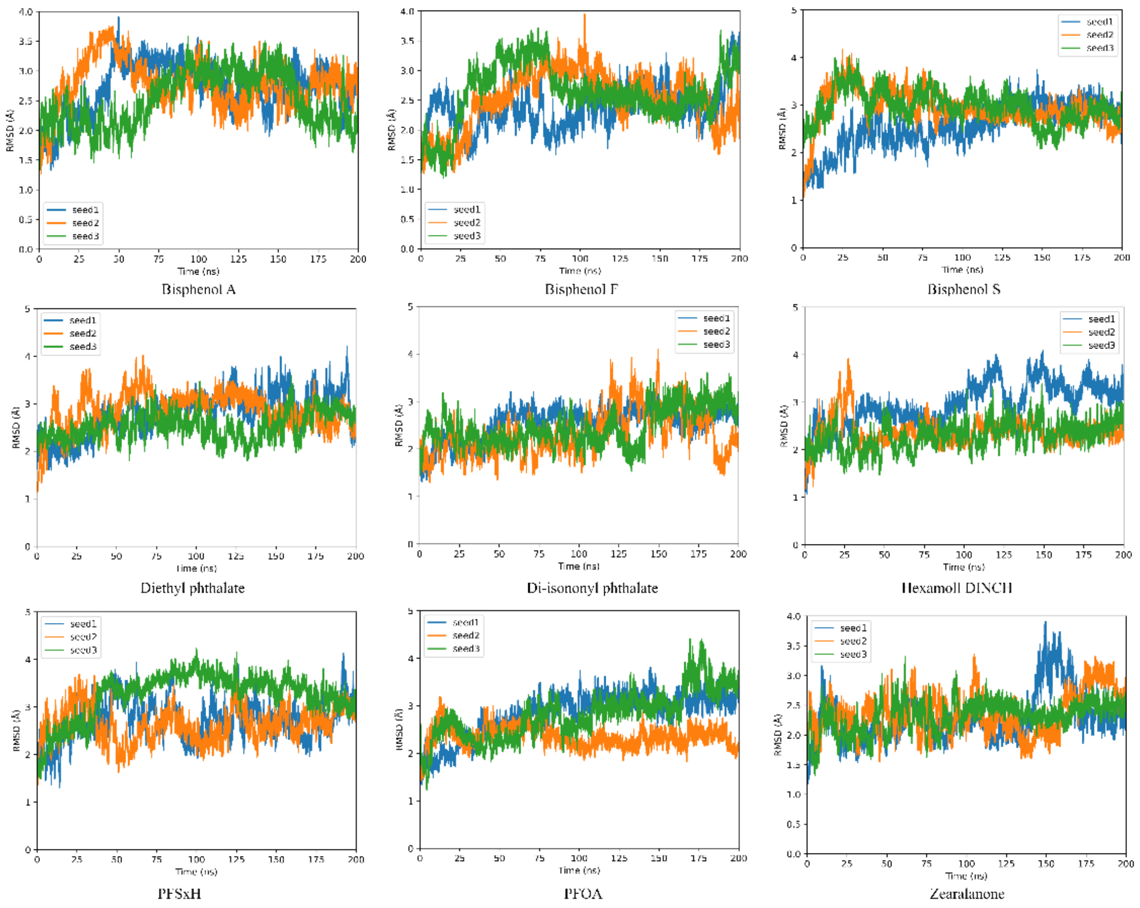

Figure S4. RMSD values, in Å, of the protein backbone atoms (C, Cα, N, O) in the complexes of ERα with the EDCs studied. The RMSD was calculated in respect to the initial docked conformation for all seeds.

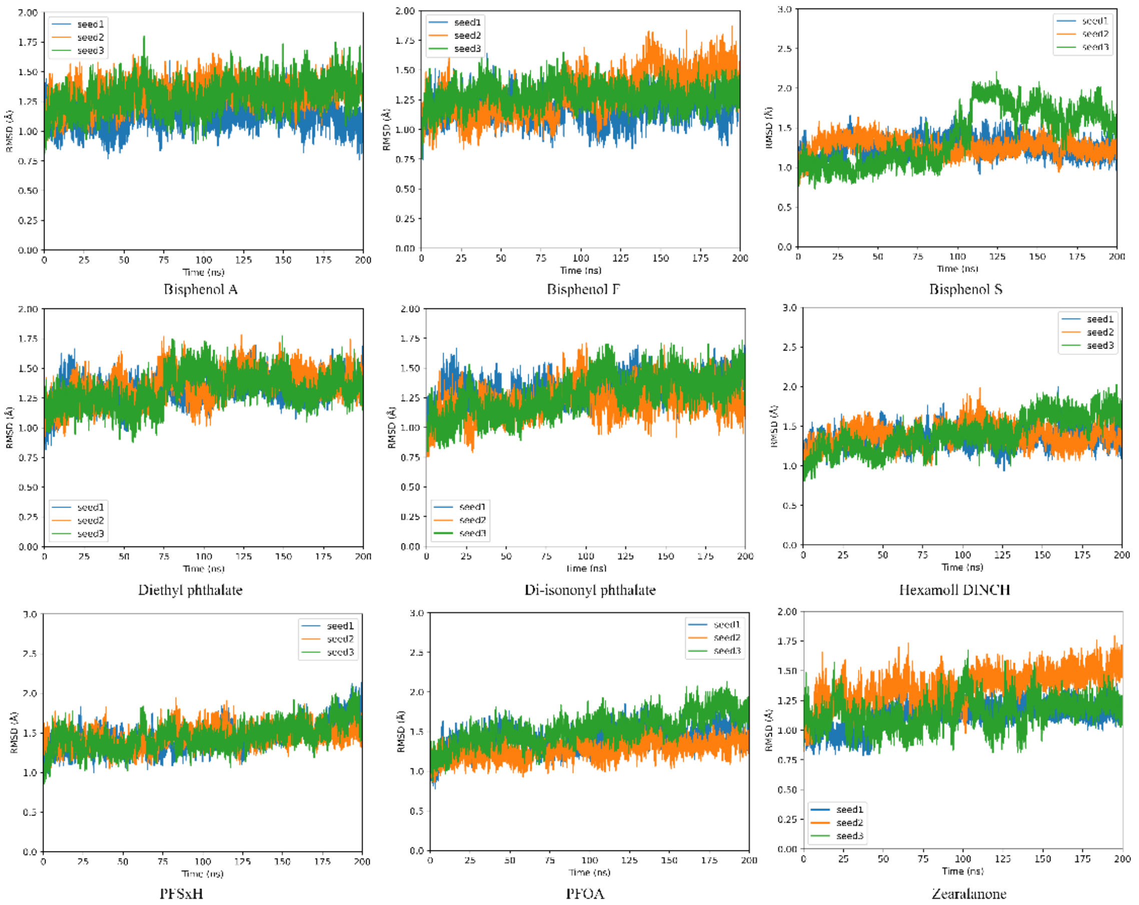

Figure S5. RMSD values, in Å, of the protein backbone atoms (C, Cα, N, O) in the complexes of AR with EDCs. The RMSD was calculated in respect to the initial docked conformation structure for all seeds.

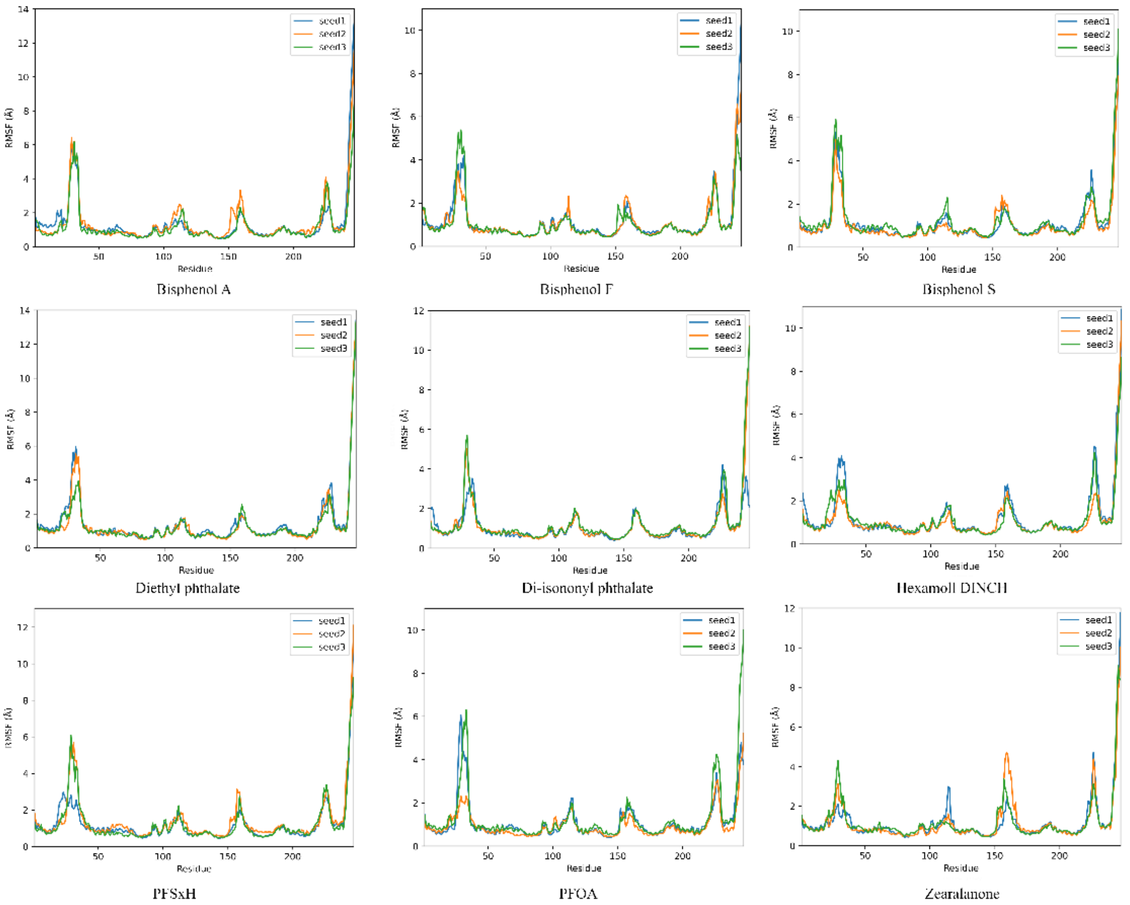

Figure S6. Atomic fluctuations of the receptor residues in the different complexes for ERα in all seeds. The RMSF was calculated on Cα atoms relative to the initial docked conformation structure.

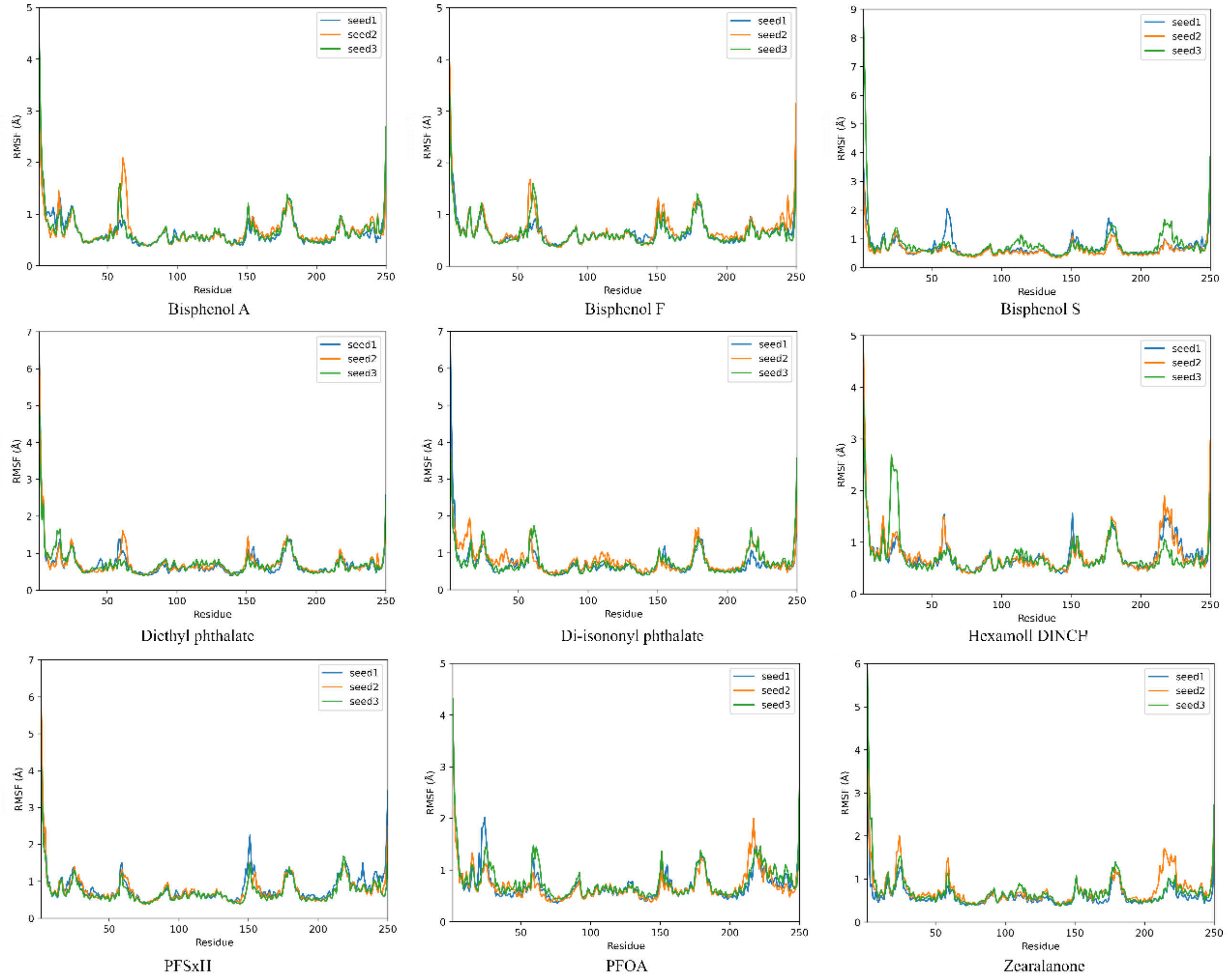

Figure S7. Atomic fluctuations of the receptor residues in the different complexes for AR in all seeds. The RMSF was calculated on Cα atoms relative to the initial docked conformation structure.

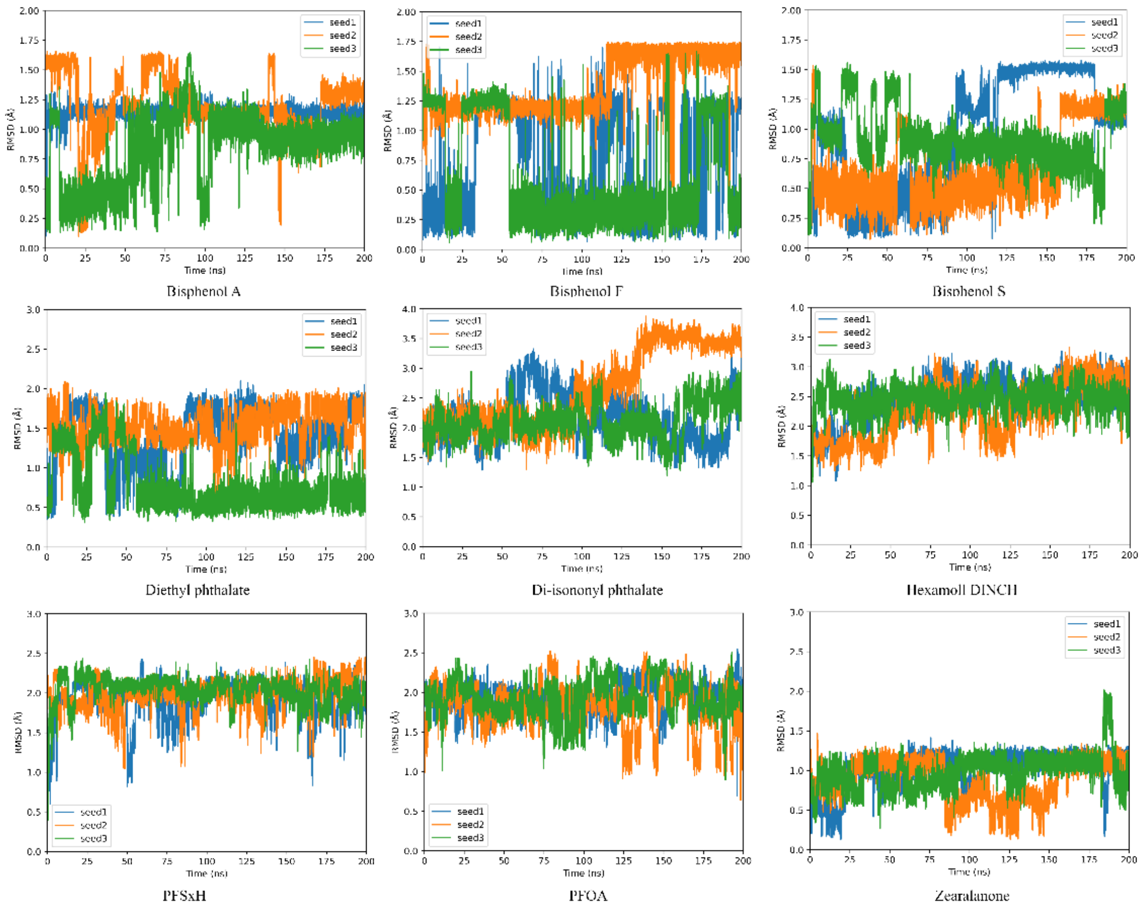

Figure S8. RMSD values, in Å, of the ligands in the complexes of ERα. The RMSD was calculated in respect to the initial docked conformation structure for all seeds.

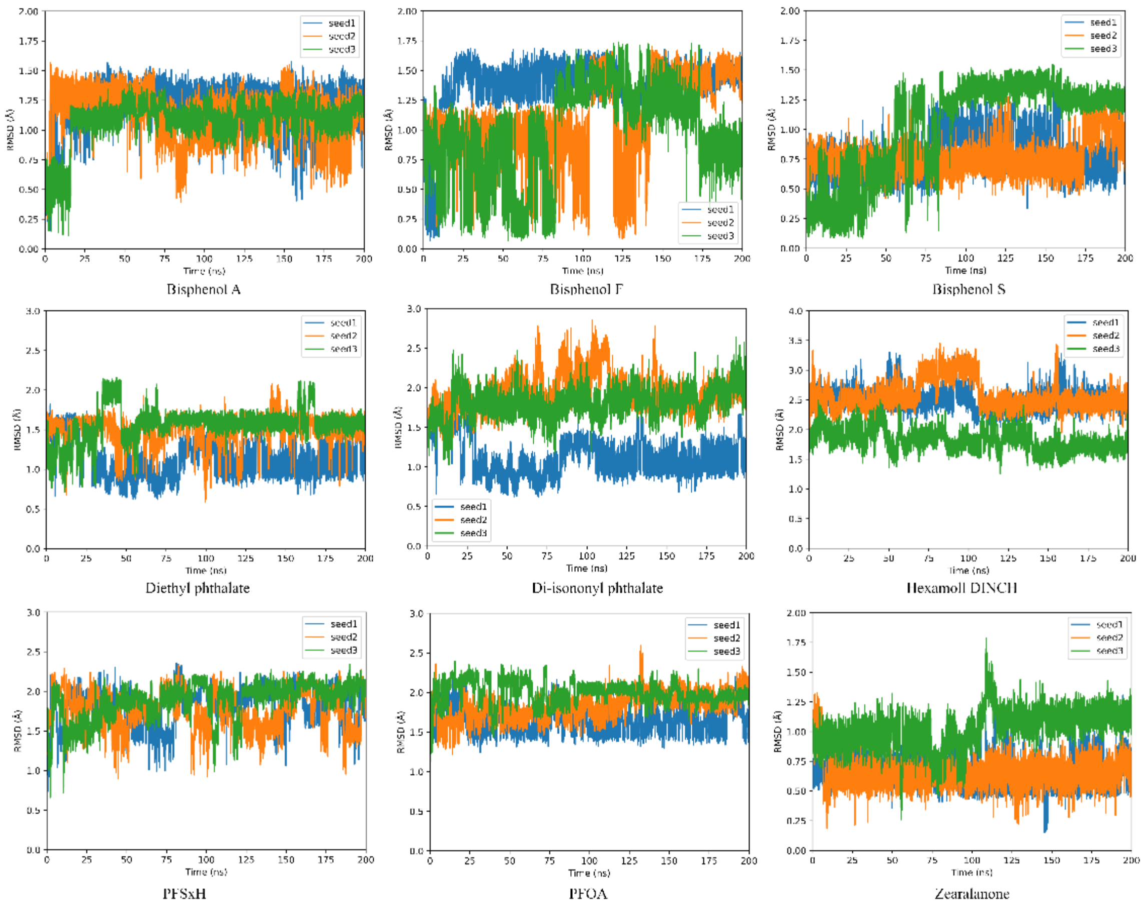

Figure S9. RMSD values, in Å, of the ligands in the complexes of AR. The RMSD was calculated in respect to the initial docked conformation structure for all seeds.

Table S2. Hydrogen bonds present in the complexes of ERα.

EDC	Residues	% of appearance
Bisphenol A	Glu48	32/29[a]
	His219	84
Bisphenol F	Glu48	71/17[a]
	His219	82
Bisphenol S	Glu48	51/18[a]
	Glu114	10
	His219	79
Diethyl phthalate	His219	10
Di-isononyl phthalate	ND[b]	ND
Hexamoll DINCH	ND	ND
Perfluorohexane-1-sulphonic acid	Arg89	15
Perfluoro-octanoic acid	His219	31/12[a]
	Lys226	10
Zearalenone	Thr42	36
	Met223	20
	Asn227	14

[a]Hydrogen bond between the same atoms appearing on more than one occasion

[b]ND=No direct interaction

Table S3. Hydrogen bonds present in the complexes of AR.

EDC	Residues	% of appearance
Bisphenol A	Asn37<br>Gln43	88<br>12/5[a]
Bisphenol F	Asn37<br>Gln43<br>Thr209	90<br>35/10[a]<br>33
Bisphenol S	Asn37<br>Gln43	77<br>43
Diethyl phthalate	Arg84	72/41[a]
Di-isononyl phthalate	Arg84	73/41[a]
Hexamoll DINCH	ND	ND
Perfluorohexane-1-sulphonic acid	Asn37<br>Thr209	29/20/18[a]<br>34/25/17[a]
Perfluoro-octanoic acid	Asn37<br>Trh209	75<br>74
Zearalenone	Gln43<br>Ser110	31<br>55

[a]Hydrogen bond between the same atoms appearing on more than one occasion

[b]ND=No direct interaction

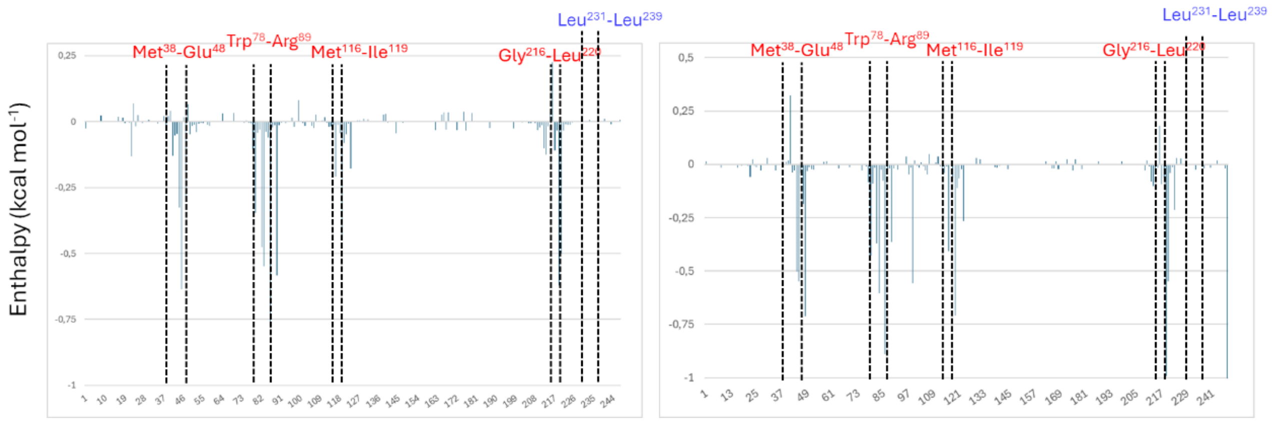

Figure S10. Per residue decomposition of the enthalpy based on the MMGBSA calculations for all the residues in the ERα complexes with bisphenol A (left) and F (right). All units are in kcal mol^{-1}.

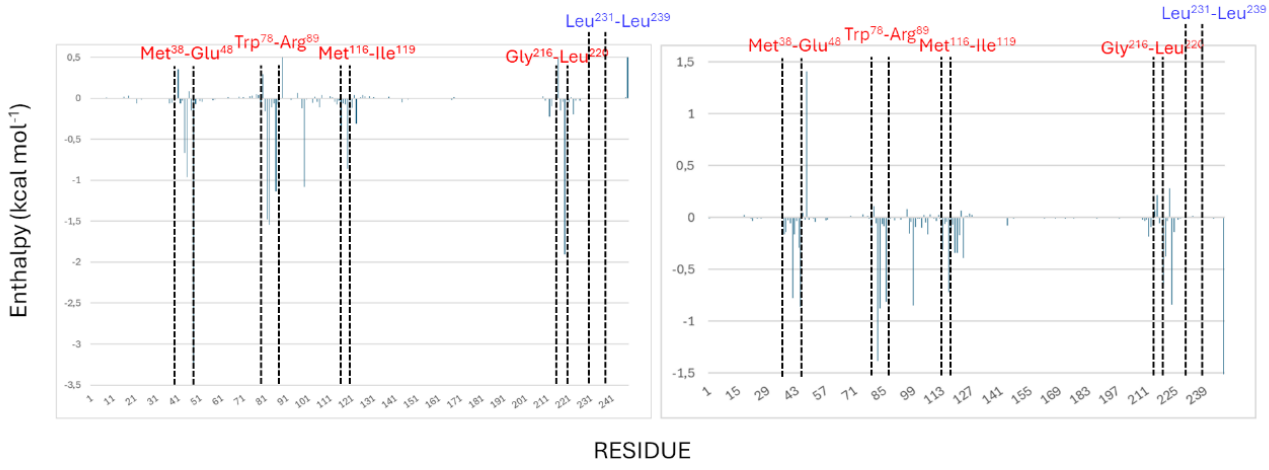

Figure S11. Per residue decomposition of the enthalpy based on the MMGBSA calculations for all the residues in the ERα complexes with bisphenol S (left) and diethyl phthalate (right). All units are in kcal mol^{-1}.

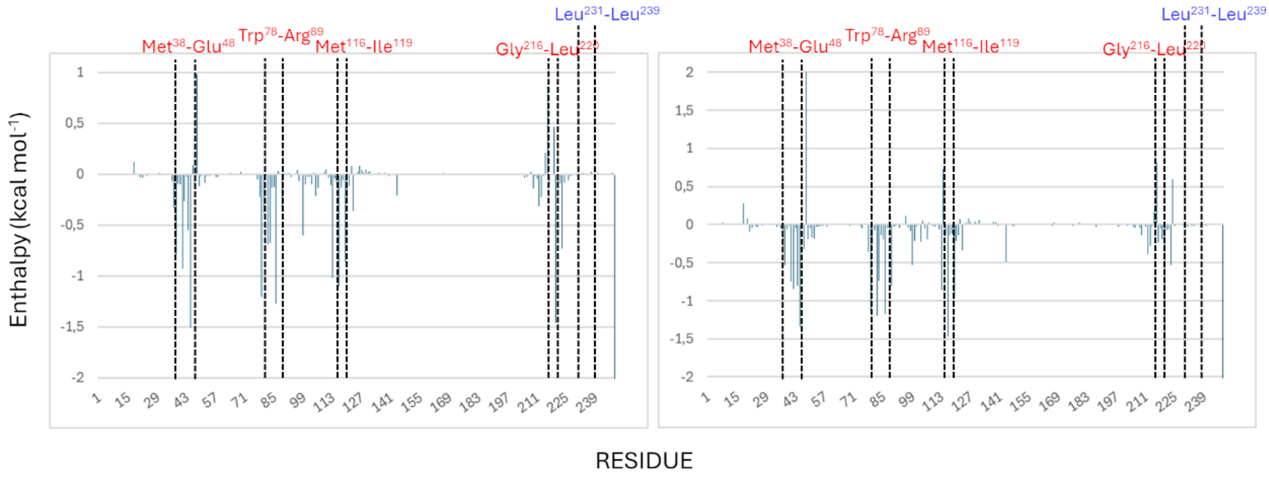

Figure S12. Per residue decomposition of the enthalpy based on the MMGBSA calculations for all the residues in the ERα diethylphthalate complexes with diisononyl phthalate (left) and Hexamoll DINCH (right). All units are in kcal mol^{-1}.

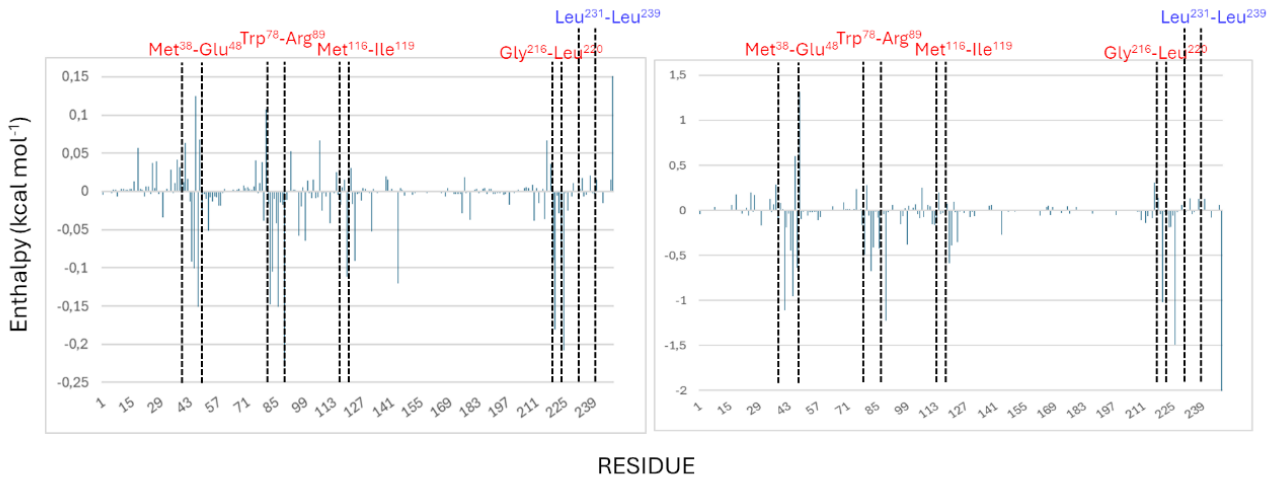

Figure S13. Per residue decomposition of the enthalpy based on the MMGBSA calculations for all the residues in the ERα complexes with PFHxS (left) and PFOA (right). All units are in kcal mol^{-1}.

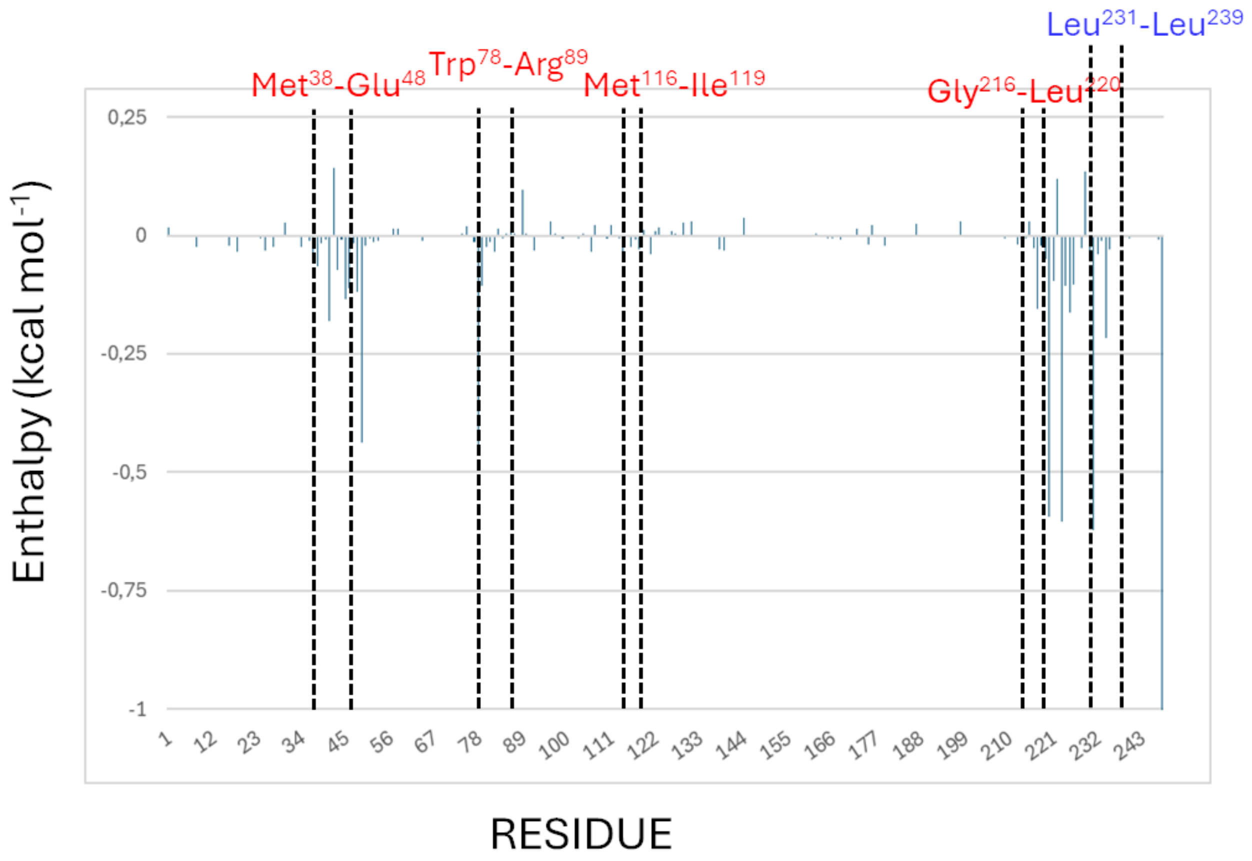

Figure S14. Per residue decomposition of the enthalpy based on the MMGBSA calculations for all the residues in the ERα complex with zearalanone. All units are in kcal mol^{-1}.

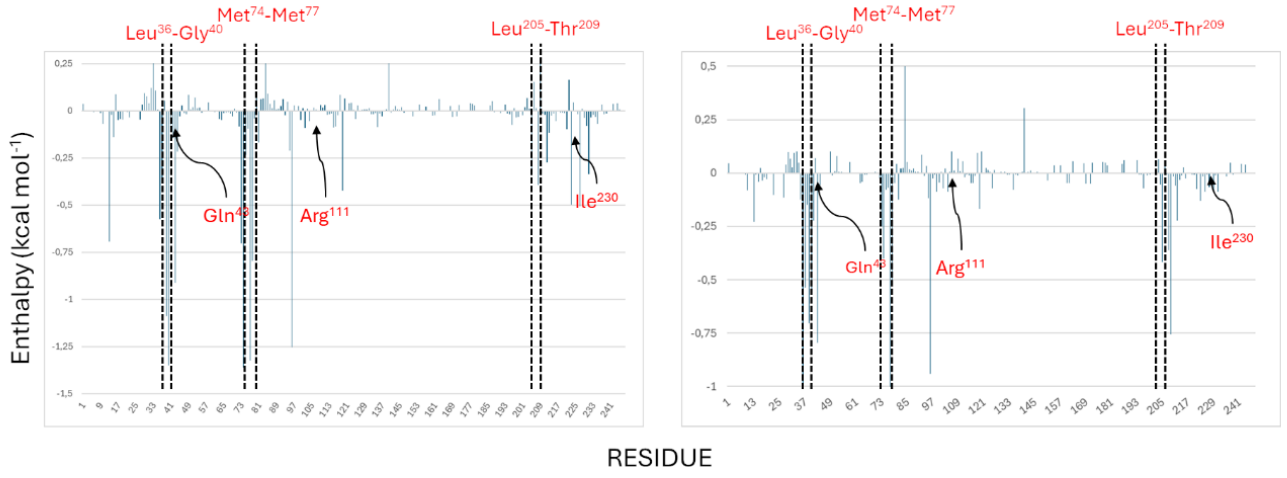

Figure S15. Per residue decomposition of the enthalpy based on the MMGBSA calculations for all the residues in the AR complexes with bisphenol A (left) and F (right). All units are in kcal mol^{-1}.

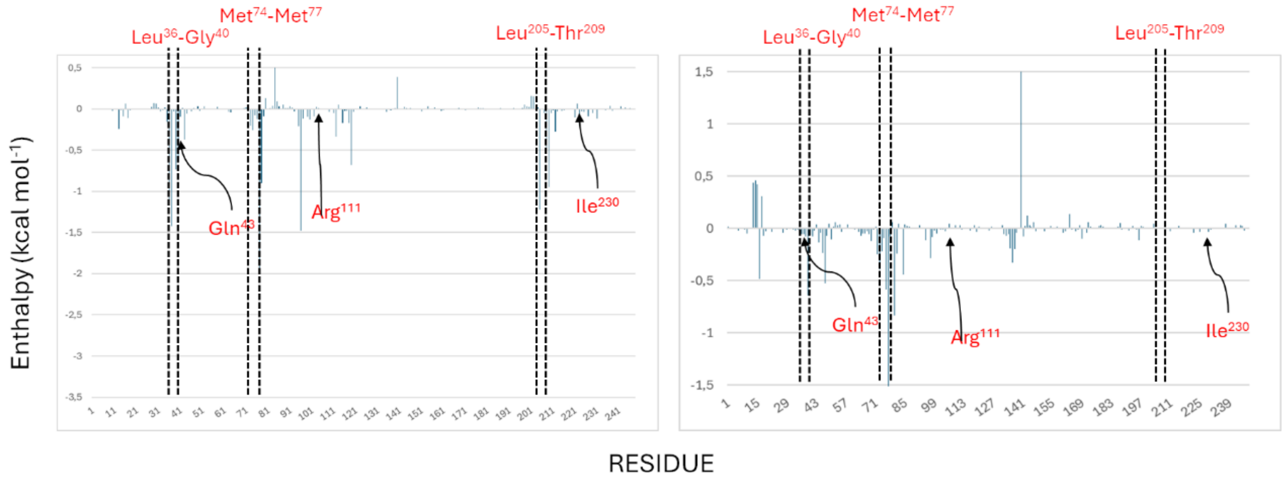

Figure S16. Per residue decomposition of the enthalpy based on the MMGBSA calculations for all the residues in the AR complexes with bisphenol S (left) and diethyl phthalate (right). All units are in kcal mol^{-1}.

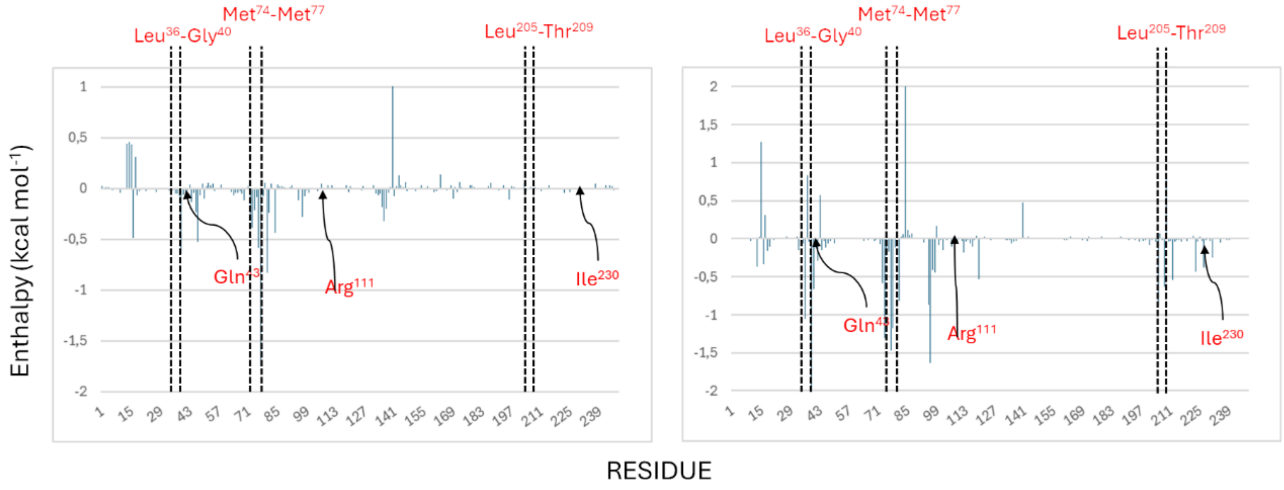

Figure S17. Per residue decomposition of the enthalpy based on the MMGBSA calculations for all the residues in the AR complexes with diisononyl phthalate (left) and Hexamoll DINCH (right). All units are in kcal mol^{-1}.

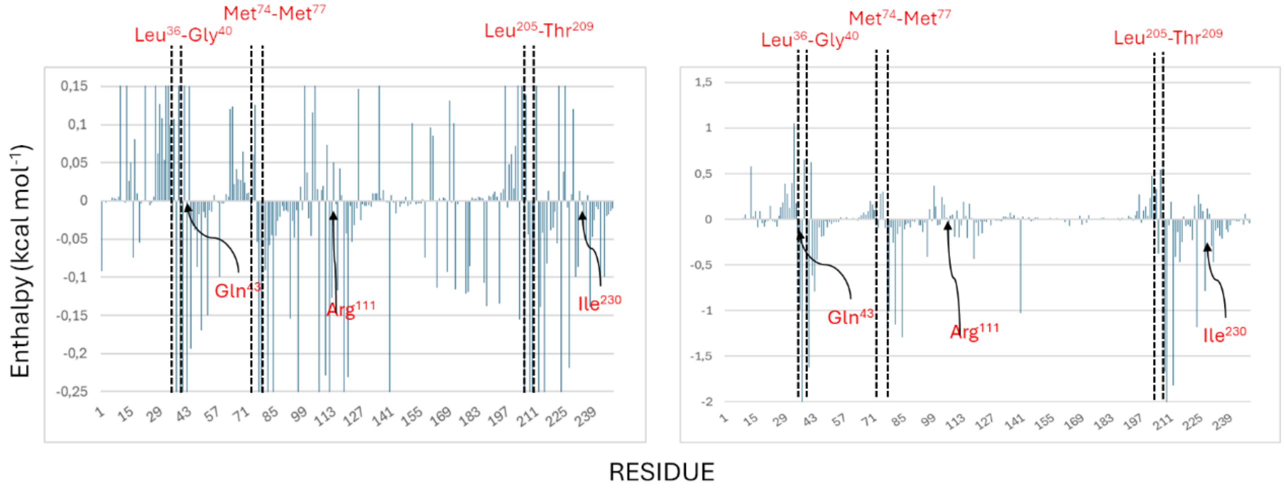

Figure S18. Per residue decomposition of the enthalpy based on the MM-GBSA calculations for all the residues in the AR complexes with PFHxS (left) and PFOA (right). All units are in kcal mol^{-1}.

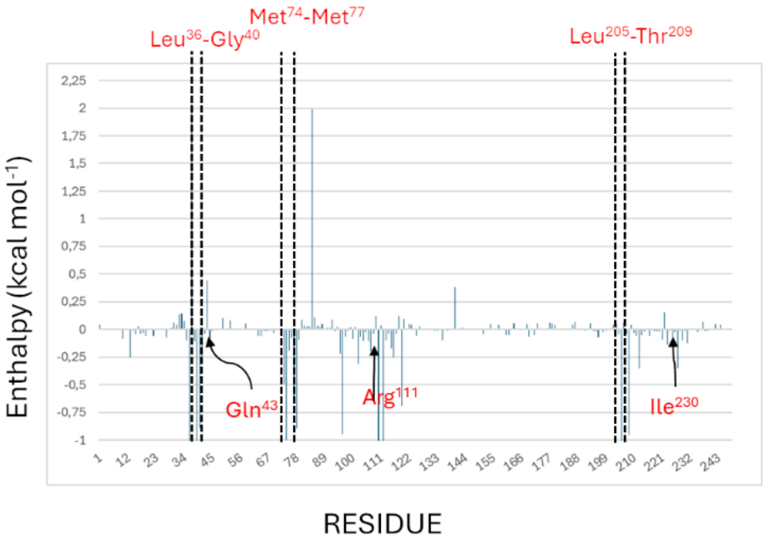

Figure S19. Per residue decomposition of the enthalpy based on the MMGBSA calculations for all the residues in the AR complex with zearalanone. All units are in kcal mol^{-1}.

Table S4. MM-GBSA binding affinity calculations for the EDCs in complex with ERα. Standard error of mean values are included in parentheses.

**EDC**	**Binding energy (kcal mol^{-1})**								
	**vdW**	**Eel**	**E_{GB}**	**$E_{nonpolar}$**	**ΔG_{gas}**	**ΔG_{solv}**	**ΔH_{total}**	**TΔS**	**ΔG^a**
Bisphenol A	-17.82 (0.54)	-13.09 (0.44)	17.03 (0.54)	-2.69 (0.08)	-30.91 (0.97)	14.34 (0.46)	-16.57 (0.51)	-8.69 (1.24)	-7.88 (0.61)
Bisphenol F	-20.49 (0.41)	-18.69 (0.37)	23.91 (0.47)	-3.27 (0.07)	-39.18 (0.77)	20.64 (0.40)	-18.55 (0.37)	-11.63 (1.30)	-6.92 (0.53)
Bisphenol S	-29.97 (0.08)	-27.57 (0.12)	38.39 (0.07)	-5.11 (0.00)	-57.53 (0.09)	33.27 (0.07)	-24.26 (0.06)	-18.89 (0.41)	-5.37 (0.14)
Diethyl phthalate	-34.22 (0.08)	-8.79 (0.10)	15.48 (0.07)	-5.02 (0.01)	-43.01 (0.17)	10.45 (0.06)	-32.56 (0.11)	-18.23 (0.55)	-14.33 (0.20)
Diisononyl phthalate	-60.49 (0.10)	-0.88 (0.05)	14.27 (0.06)	-8.15 (0.01)	-61.37 (0.12)	6.13 (0.06)	-55.24 (0.10)	-25.87 (0.40)	-29.37 (0.15)
Hexamoll DINCH	-61.95 (0.08)	4.12 (0.04)	9.68 (0.04)	-8.09 (0.01)	-57.83 (0.09)	1.59 (0.04)	-56.23 (0.08)	-25.28 (0.53)	-30.95 (0.18)
Perfluoro-octanoic acid	-25.46 (0.17)	55.60 (0.49)	-46.23 (0.44)	-4.79 (0.03)	30.14 (0.49)	-51.02 (0.44)	-20.87 (0.16)	-17.34 (0.58)	-3.53 (0.23)
Perfluorohexane-1-sulphonic acid	-3.71 (0.33)	40.79 (0.42)	-38.63 (0.28)	-0.66 (0.06)	37.08 (0.14)	-39.28 (0.33)	-2.20 (0.23)	0.43 (0.91)	-1.77 (0.35)
Zearalenone	-19.72 (0.55)	-5.88 (0.24)	11.93 (0.38)	-2.62 (0.07)	-25.60 (0.76)	9.32 (0.31)	-16.29 (0.47)	-9.20 (1.16)	-7.09 (0.57)

[a] ΔG= ΔH - TΔS

Table S5. MM-GBSA binding affinity calculations for the EDCs in complex with AR. Standard error of mean values are included in parentheses.

**EDC**	**Binding energy (kcal mol^{-1})**								
	**vdW**	**Eel**	**E_{GB}**	**$E_{nonpolar}$**	**ΔG_{gas}**	**ΔG_{solv}**	**ΔH_{total}**	**TΔS**	**ΔG**
Bisphenol A	-33.46 (0.07)	-12.31 (0.14)	21.57 (0.11)	-5.08 (0.00)	-45.77 (0.12)	16.49 (0.11)	-29.28 (0.06)	-19.51 (0.26)	-9.77 (0.10)
Bisphenol F	-22.05 (0.42)	-15.73 (0.36)	21.08 (0.14)	-3.45 (0.07)	-37.78 (0.74)	17.63 (0.37)	-20.15 (0.39)	-12.02 (1.32)	-8.13 (0.54)
Bisphenol S	-32.66 (0.08)	-23.78 (0.14)	38.42 (0.11)	-5.26 (0.00)	-56.46 (0.13)	33.16 (0.11)	-23.30 (0.07)	-19.96 (0.30)	-3.34 (0.11)
Diethyl phthalate	-4.18 (0.37)	-1.46 (0.13)	2.21 (0.19)	0.54 (0.04)	-5.63 (0.50)	1.67 (0.14)	-3.96 (0.36)	2.76 (0.91)	-1.2 (0.98)
Diisononyl phthalate	-31.67 (0.07)	-32.01 (0.11)	32.38 (0.06)	-4.75 (0.00)	-63.68 (0.10)	-27.63 (0.06)	-36.04 (0.06)	18.98 (0.29)	-17.06 (0.10)
Hexamoll DINCH	-70.73 (0.87)	-8.25 (0.06)	23.96 (0.05)	-9.32 (0.01)	-78.98 (0.09)	14.64 (0.05)	-64.34 (0.09)	-27.46 (0.41)	-36.88 (0.15)
Perfluoro-octanoic acid	-28.68 (0.27)	-63.12 (0.30)	66.01 (0.31)	-5.45 (0.05)	-91.81 (0.53)	60.56 (0.27)	-31.25 (0.29)	-19.14 (0.73)	-12.11 (0.78)
Perfluorohexane-1-sulphonic acid	-18.11 (0.53)	-41.82 (0.51)	45.67 (0.56)	-3.37 (0.09)	-59.93 (0.87)	42.29 (0.51)	-17.64 (0.51)	-9.43 (1.18)	-8.21 (0.60)
Zearalenone	-45.94 (0.08)	-12.59 (0.12)	26.27 (0.08)	-6.40 (0.01)	-58.53 (0.13)	19.87 (0.08)	-38.66 (0.08)	-20.64 (0.33)	-18.02 (0.33)

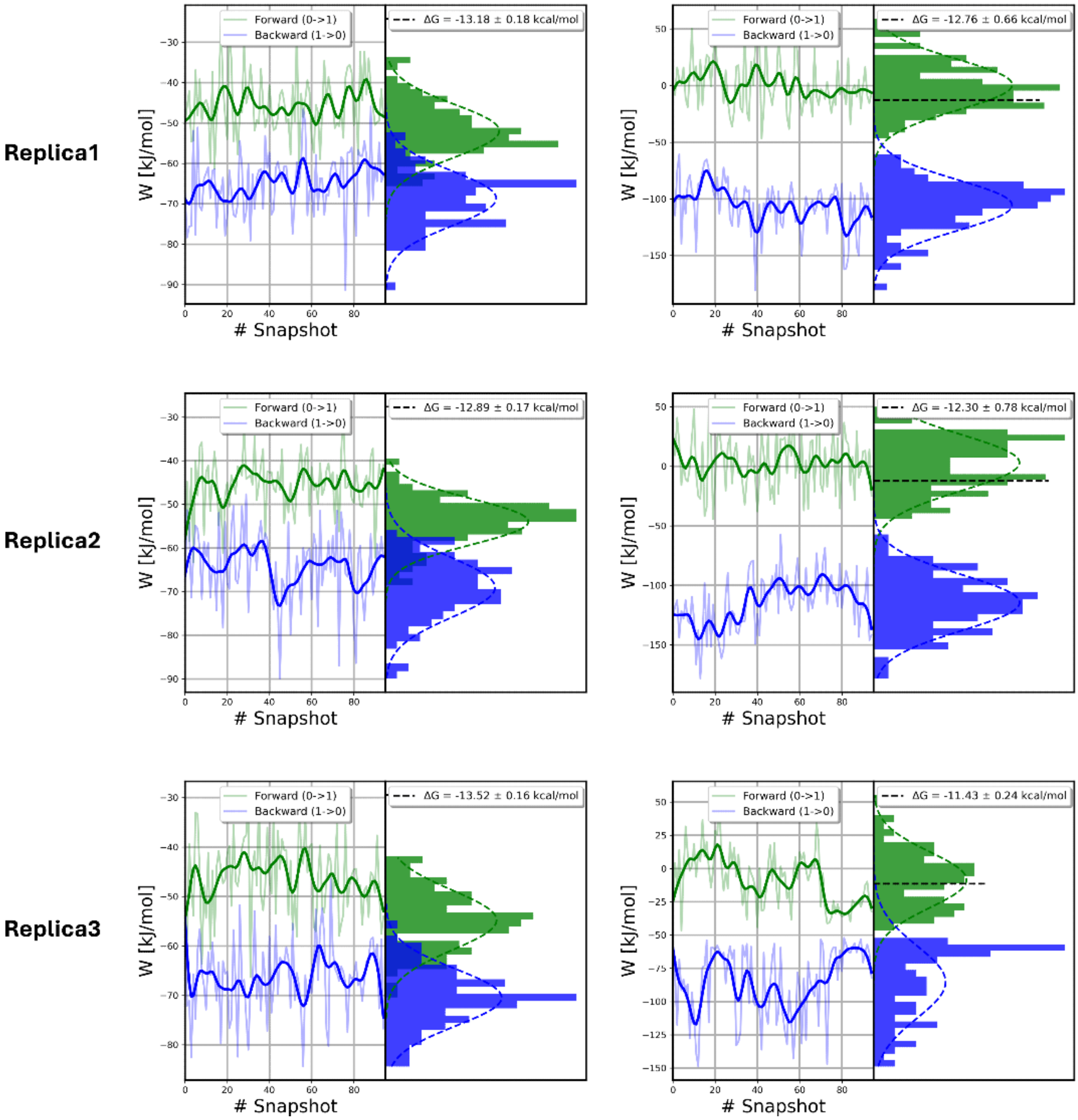

Figure S20. Alchemical relative free energy binding calculation results for the bisphenol A to bisphenol F transition. Three replicas were performed for each transition. The left panels represent the transition in the solvent and the right panels represent the same transition in the complex with the ERα.

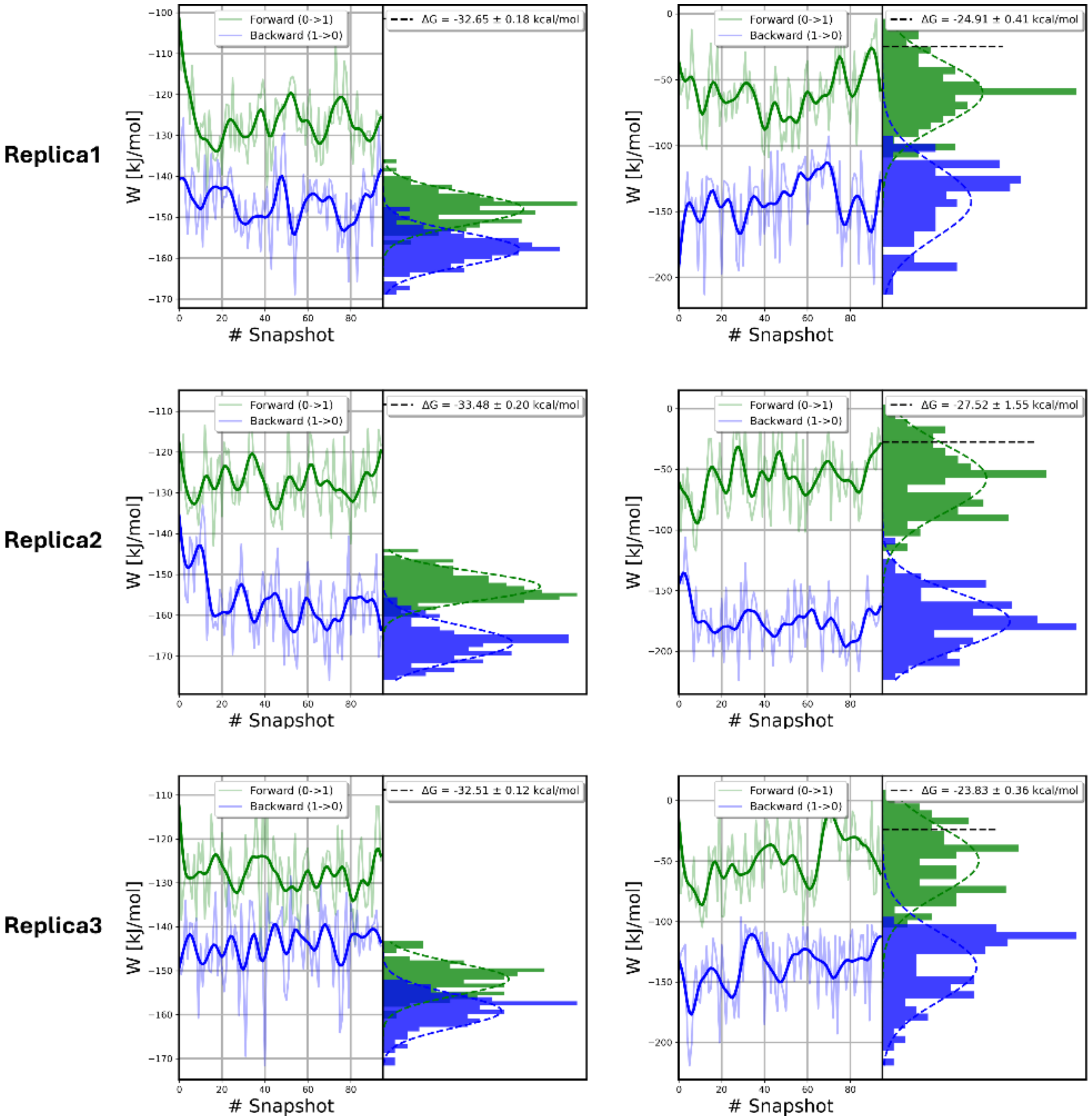

Figure S21. Alchemical relative free energy binding calculation results for the bisphenol A to bisphenol S transition. Three replicas were performed for each transition. The left panels represent the transition in the solvent and the right panels represent the same transition in the complex with the ERα.

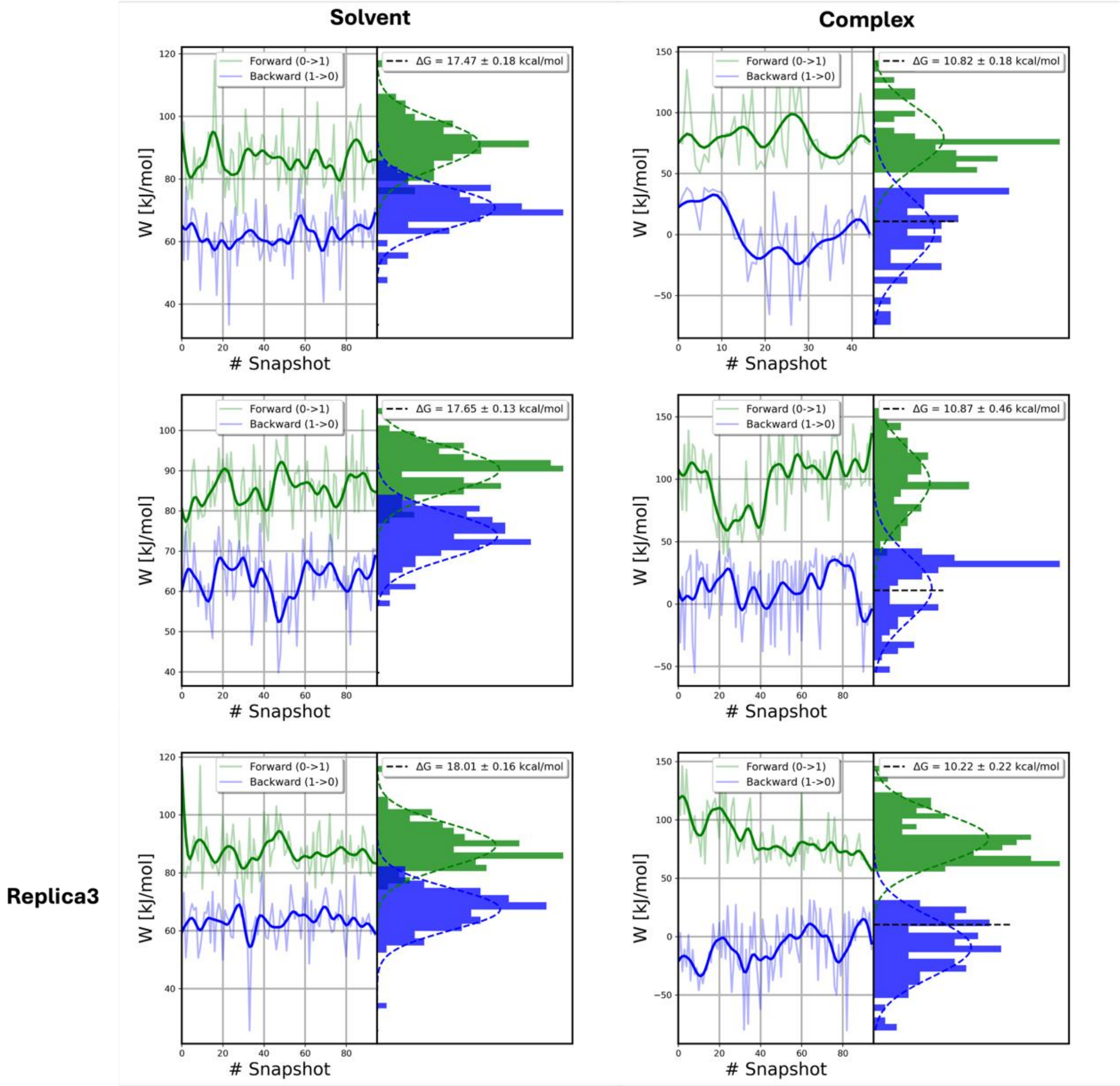

Figure S22. Alchemical relative free energy binding calculation results for the bisphenol S to bisphenol F transition. Three replicas were performed for each transition. The left panels represent the transition in the solvent and the right panels represent the same transition in the complex with the ERα.

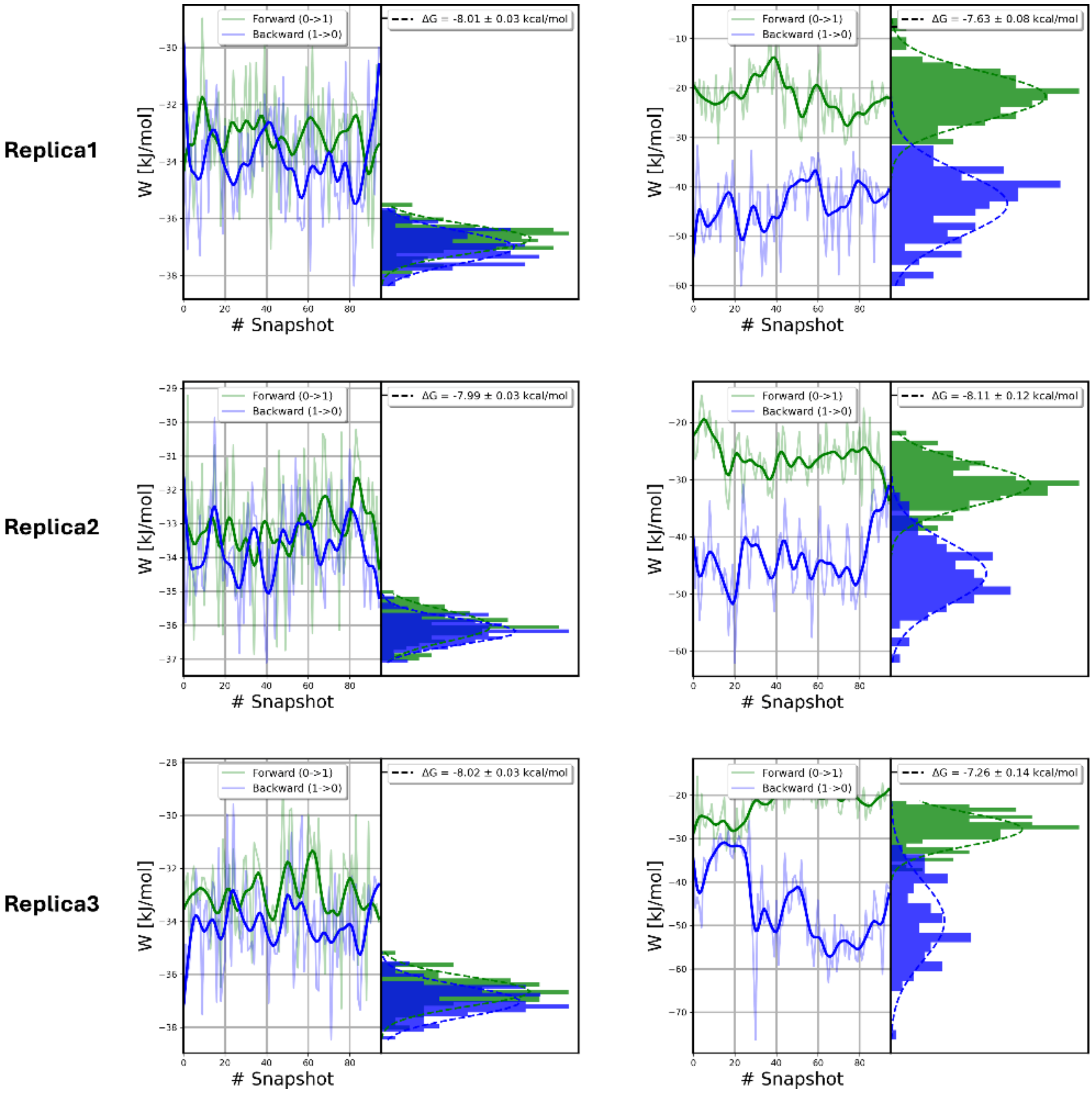

Figure S23. Alchemical relative free energy binding calculation results for the bisphenol A to bisphenol F transition. Three replicas were performed for each transition. The left panels represent the transition in the solvent and the right panels represent the same transition in the complex with the AR.

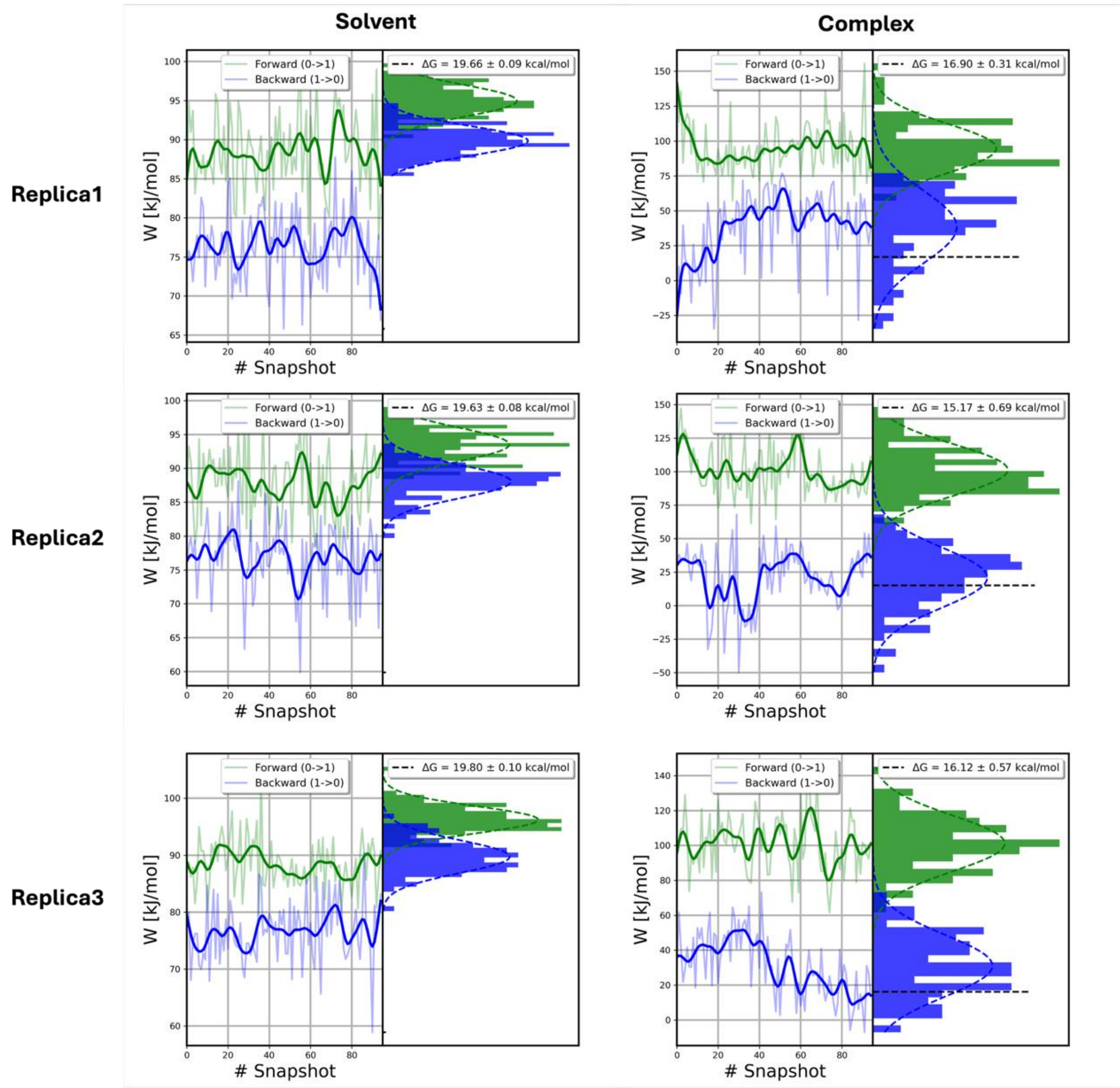

Figure S24. Alchemical relative free energy binding calculation results for the bisphenol A to bisphenol S transition. Three replicas were performed for each transition. The left panels represent the transition in the solvent and the right panels represent the same transition in the complex with the AR.

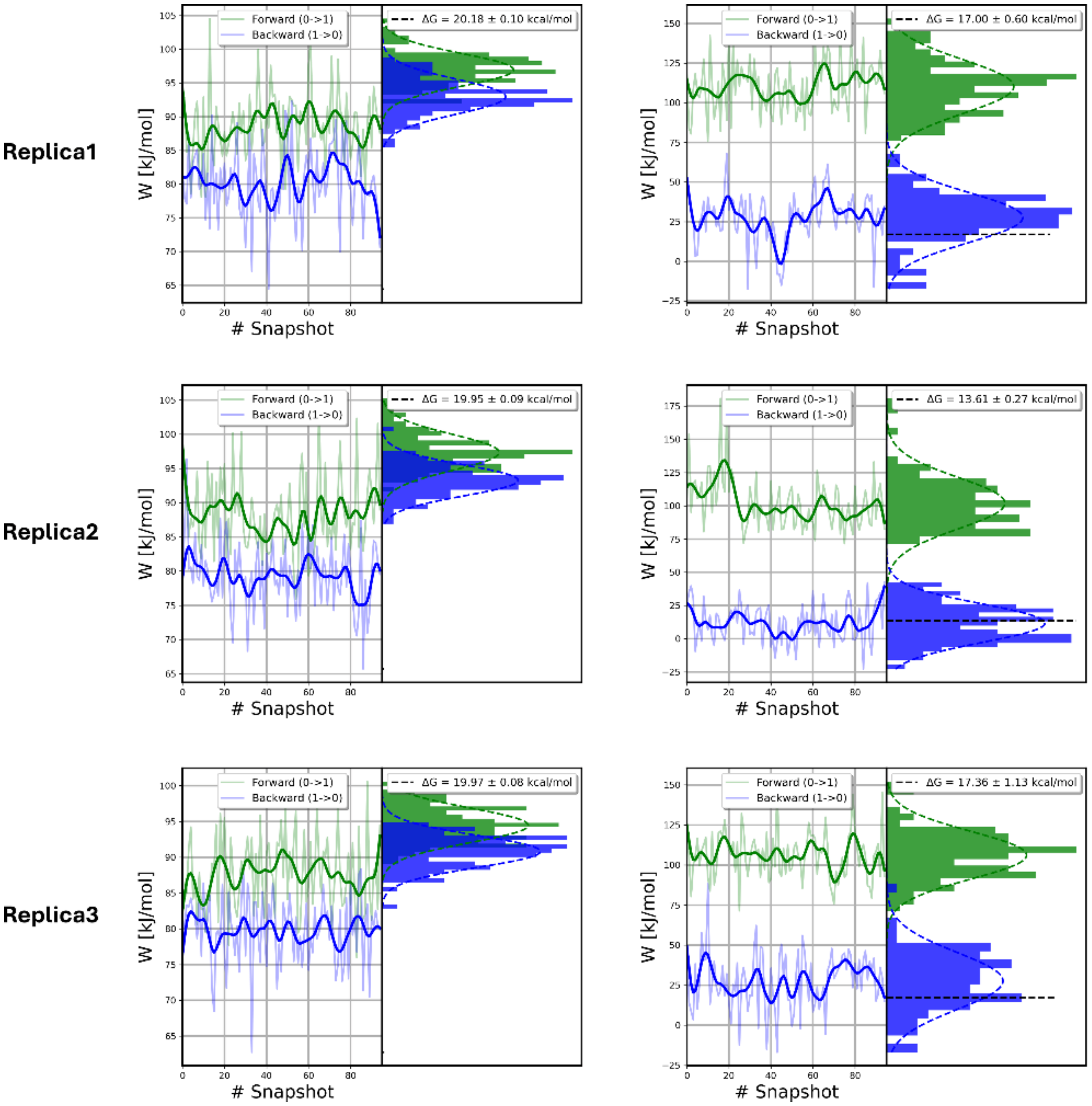

Figure S25. Alchemical relative free energy binding calculation results for the bisphenol S to bisphenol F transition. Three replicas were performed for each transition. The left panels represent the transition in the solvent and the right panels represent the same transition in the complex with the AR.

References

(1) Shiau, A. K.; Barstad, D.; Loria, P. M.; Cheng, L.; Kushner, P. J.; Agard, D. A.; Greene, G. L. The Structural Basis of Estrogen Receptor/Coactivator Recognition and the Antagonism of This Interaction by Tamoxifen. *Cell* **1998**, *95* (7), 927–937. https://doi.org/10.1016/S0092-8674(00)81717-1.

(2) Hur, E.; Pfaff, S. J.; Payne, E. S.; Grøn, H.; Buehrer, B. M.; Fletterick, R. J. Recognition and Accommodation at the Androgen Receptor Coactivator Binding Interface. *PLoS Biol* **2004**, *2* (9), e274. https://doi.org/10.1371/journal.pbio.0020274.

(3) Eastman, P.; Swails, J.; Chodera, J. D.; McGibbon, R. T.; Zhao, Y.; Beauchamp, K. A.; Wang, L.-P.; Simmonett, A. C.; Harrigan, M. P.; Stern, C. D.; Wiewiora, R. P.; Brooks, B. R.; Pande, V. S. OpenMM 7: Rapid Development of High Performance Algorithms for Molecular Dynamics. *PLoS Comput Biol* **2017**, *13* (7), e1005659. https://doi.org/10.1371/journal.pcbi.1005659.

(4) Kim, S.; Chen, J.; Cheng, T.; Gindulyte, A.; He, J.; He, S.; Li, Q.; Shoemaker, B. A.; Thiessen, P. A.; Yu, B.; Zaslavsky, L.; Zhang, J.; Bolton, E. E. PubChem 2025 Update. *Nucleic Acids Research* **2025**, *53* (D1), D1516–D1525. https://doi.org/10.1093/nar/gkae1059.

(5) O'Boyle, N. M.; Banck, M.; James, C. A.; Morley, C.; Vandermeersch, T.; Hutchison, G. R. Open Babel: An Open Chemical Toolbox. *J Cheminform* **2011**, *3* (1), 33. https://doi.org/10.1186/1758-2946-3-33.

(6) Stylianaki, E.-A.; Mouchlis, V. D.; Magkrioti, C.; Papavasileiou, K. D.; Afantitis, A.; Matralis, A. N.; Aidinis, V. Identification of Two Novel Chemical Classes of Autotaxin (ATX) Inhibitors Using Enalos Asclepios KNIME Nodes. *Bioorganic & Medicinal Chemistry Letters* **2024**, *103*, 129690. https://doi.org/10.1016/j.bmcl.2024.129690.

(7) Papadopoulou, D.; Drakopoulos, A.; Lagarias, P.; Melagraki, G.; Kollias, G.; Afantitis, A. In Silico Identification and Evaluation of Natural Products as Potential Tumor Necrosis Factor Function Inhibitors Using Advanced Enalos Asclepios KNIME Nodes. *IJMS* **2021**, *22* (19), 10220. https://doi.org/10.3390/ijms221910220.

(8) Papavasileiou, K. D.; Tsoumanis, A. C.; Lagarias, P. I.; Kolokathis, P. D.; Koutroumpa, N.-M.; Melagraki, G.; Afantitis, A. PFAS-Biomolecule Interactions: Case Study Using Asclepios Nodes and Automated Workflows in KNIME for Drug Discovery and Toxicology. In *Computational Toxicology*; Nicolotti, O., Ed.; Methods in Molecular Biology; Springer US: New York, NY, 2025; Vol. 2834, pp 393–441. https://doi.org/10.1007/978-1-0716-4003-6_19.

(9) Trott, O.; Olson, A. J. AutoDock Vina: Improving the Speed and Accuracy of Docking with a New Scoring Function, Efficient Optimization, and Multithreading. *J Comput Chem* **2010**, *31* (2), 455–461. https://doi.org/10.1002/jcc.21334.

(10) Ravindranath, P. A.; Forli, S.; Goodsell, D. S.; Olson, A. J.; Sanner, M. F. AutoDockFR: Advances in Protein-Ligand Docking with Explicitly Specified Binding Site Flexibility. *PLoS Comput Biol* **2015**, *11* (12), e1004586. https://doi.org/10.1371/journal.pcbi.1004586.

(11) Singh, U. C.; Kollman, P. A. An Approach to Computing Electrostatic Charges for Molecules. *J Comput Chem* **1984**, *5* (2), 129–145. https://doi.org/10.1002/jcc.540050204.

(12) Gasteiger, J.; Marsili, M. Iterative Partial Equalization of Orbital Electronegativity—a Rapid Access to Atomic Charges. *Tetrahedron* **1980**, *36* (22), 3219–3228. https://doi.org/10.1016/0040-4020(80)80168-2.

(13) Stephens, P. J.; Devlin, F. J.; Chabalowski, C. F.; Frisch, M. J. Ab Initio Calculation of Vibrational Absorption and Circular Dichroism Spectra Using Density Functional Force Fields. *J. Phys. Chem.* **1994**, *98* (45), 11623–11627. https://doi.org/10.1021/j100096a001.

(14) Becke, A. D. Density-Functional Thermochemistry. III. The Role of Exact Exchange. *The Journal of Chemical Physics* **1993**, *98* (7), 5648–5652. https://doi.org/10.1063/1.464913.

(15) Hehre, W. J.; Ditchfield, R.; Pople, J. A. Self—Consistent Molecular Orbital Methods. XII. Further Extensions of Gaussian—Type Basis Sets for Use in Molecular Orbital Studies of Organic Molecules. *The Journal of Chemical Physics* **1972**, *56* (5), 2257–2261. https://doi.org/10.1063/1.1677527.

(16) Barca, G. M. J.; Bertoni, C.; Carrington, L.; Datta, D.; De Silva, N.; Deustua, J. E.; Fedorov, D. G.; Gour, J. R.; Gunina, A. O.; Guidez, E.; Harville, T.; Irle, S.; Ivanic, J.; Kowalski, K.; Leang, S. S.; Li, H.; Li, W.; Lutz, J. J.; Magoulas, I.; Mato, J.; Mironov, V.; Nakata, H.; Pham, B. Q.; Piecuch, P.; Poole, D.; Pruitt, S. R.; Rendell, A. P.; Roskop, L. B.; Ruedenberg, K.; Sattasathuchana, T.; Schmidt, M. W.; Shen, J.; Slipchenko, L.; Sosonkina, M.; Sundriyal, V.; Tiwari, A.; Galvez Vallejo, J. L.; Westheimer, B.; Włoch, M.; Xu, P.; Zahariev, F.; Gordon, M. S. Recent Developments in the General Atomic and Molecular Electronic Structure System. *The Journal of Chemical Physics* **2020**, *152* (15), 154102. https://doi.org/10.1063/5.0005188.

(17) Bayly, C. I.; Cieplak, P.; Cornell, W.; Kollman, P. A. A Well-Behaved Electrostatic Potential Based Method Using Charge Restraints for Deriving Atomic Charges: The RESP Model. *J. Phys. Chem.* **1993**, *97* (40), 10269–10280. https://doi.org/10.1021/j100142a004.

(18) Maier, J. A.; Martinez, C.; Kasavajhala, K.; Wickstrom, L.; Hauser, K. E.; Simmerling, C. ff14SB: Improving the Accuracy of Protein Side Chain and Backbone Parameters from ff99SB. *J. Chem. Theory Comput.* **2015**, *11* (8), 3696–3713. https://doi.org/10.1021/acs.jctc.5b00255.

(19) Wang, J.; Wolf, R. M.; Caldwell, J. W.; Kollman, P. A.; Case, D. A. Development and Testing of a General Amber Force Field. *J Comput Chem* **2004**, *25* (9), 1157–1174. https://doi.org/10.1002/jcc.20035.

(20) Mark, P.; Nilsson, L. Structure and Dynamics of the TIP3P, SPC, and SPC/E Water Models at 298 K. *J. Phys. Chem. A* **2001**, *105* (43), 9954–9960. https://doi.org/10.1021/jp003020w.

(21) Berendsen, H. J. C.; Grigera, J. R.; Straatsma, T. P. The Missing Term in Effective Pair Potentials. *J. Phys. Chem.* **1987**, *91* (24), 6269–6271. https://doi.org/10.1021/j100308a038.

(22) Izaguirre, J. A.; Catarello, D. P.; Wozniak, J. M.; Skeel, R. D. Langevin Stabilization of Molecular Dynamics. *The Journal of Chemical Physics* **2001**, *114* (5), 2090–2098. https://doi.org/10.1063/1.1332996.

(23) Åqvist, J.; Wennerström, P.; Nervall, M.; Bjelic, S.; Brandsdal, B. O. Molecular Dynamics Simulations of Water and Biomolecules with a Monte Carlo Constant Pressure Algorithm. *Chemical Physics Letters* **2004**, *384* (4–6), 288–294. https://doi.org/10.1016/j.cplett.2003.12.039.

(24) Chow, K.-H.; Ferguson, D. M. Isothermal-Isobaric Molecular Dynamics Simulations with Monte Carlo Volume Sampling. *Computer Physics Communications* **1995**, *91* (1–3), 283–289. https://doi.org/10.1016/0010-4655(95)00059-O.

(25) Essmann, U.; Perera, L.; Berkowitz, M. L.; Darden, T.; Lee, H.; Pedersen, L. G. A Smooth Particle Mesh Ewald Method. *The Journal of Chemical Physics* **1995**, *103* (19), 8577–8593. https://doi.org/10.1063/1.470117.

(26) Case, D. A.; Aktulga, H. M.; Belfon, K.; Cerutti, D. S.; Cisneros, G. A.; Cruzeiro, V. W. D.; Götz, A. W.; Gohlke, H.; Izadi, S.; Kasavajhala, K.; Kaymak, M. C.; King, E.; Kurtzman, T.; Nguyen, H. M.; O'Hearn, K. A.; Onufriev, A. V.; Pan, F.; Pantano, S.; Qi, R.; Rahnamoun, A.; Schott-Verdugo, S.; Wang, J.; Wei, H.; Wu, Y.; Cheatham, T. E. I.; Roe, D. R.; Roitberg, A.; Simmerling, C.; York, D. M.; Merz, K. M. Jr. AmberTools. *Amber 2021, University of California, San Frnacisco* **2021**.

(27) Roe, D. R.; Cheatham, T. E. PTRAJ and CPPTRAJ: Software for Processing and Analysis of Molecular Dynamics Trajectory Data. *J. Chem. Theory Comput.* **2013**, *9* (7), 3084–3095. https://doi.org/10.1021/ct400341p.

(28) Kollman, P. A.; Massova, I.; Reyes, C.; Kuhn, B.; Huo, S.; Chong, L.; Lee, M.; Lee, T.; Duan, Y.; Wang, W.; Donini, O.; Cieplak, P.; Srinivasan, J.; Case, D. A.; Cheatham, T. E. Calculating Structures and Free Energies of Complex Molecules: Combining Molecular Mechanics and Continuum Models. *Acc. Chem. Res.* **2000**, *33* (12), 889–897. https://doi.org/10.1021/ar000033j.

(29) Gohlke, H.; Kiel, C.; Case, D. A. Insights into Protein–Protein Binding by Binding Free Energy Calculation and Free Energy Decomposition for the Ras–Raf and Ras–RalGDS Complexes. *Journal of Molecular Biology* **2003**, *330* (4), 891–913. https://doi.org/10.1016/S0022-2836(03)00610-7.

(30) Hou, T.; Wang, J.; Li, Y.; Wang, W. Assessing the Performance of the MM/PBSA and MM/GBSA Methods. 1. The Accuracy of Binding Free Energy Calculations Based on Molecular Dynamics Simulations. *J. Chem. Inf. Model.* **2011**, *51* (1), 69–82. https://doi.org/10.1021/ci100275a.

(31) Pettersen, E. F.; Goddard, T. D.; Huang, C. C.; Couch, G. S.; Greenblatt, D. M.; Meng, E. C.; Ferrin, T. E. UCSF Chimera—A Visualization System for Exploratory Research and Analysis. *J Comput Chem* **2004**, *25* (13), 1605–1612. https://doi.org/10.1002/jcc.20084.

(32) Gapsys, V.; Pérez-Benito, L.; Aldeghi, M.; Seeliger, D.; Van Vlijmen, H.; Tresadern, G.; De Groot, B. L. Large Scale Relative Protein Ligand Binding Affinities Using Non-Equilibrium Alchemy. *Chem. Sci.* **2020**, *11* (4), 1140–1152. https://doi.org/10.1039/C9SC03754C.

(33) Gapsys, V.; Yildirim, A.; Aldeghi, M.; Khalak, Y.; Van Der Spoel, D.; De Groot, B. L. Accurate Absolute Free Energies for Ligand–Protein Binding Based on Non-Equilibrium Approaches. *Commun Chem* **2021**, *4* (1), 61. https://doi.org/10.1038/s42004-021-00498-y.

(34) Khalak, Y.; Tresadern, G.; Aldeghi, M.; Baumann, H. M.; Mobley, D. L.; De Groot, B. L.; Gapsys, V. Alchemical Absolute Protein–Ligand Binding Free Energies for Drug Design. *Chem. Sci.* **2021**, *12* (41), 13958–13971. https://doi.org/10.1039/D1SC03472C.

(35) Gapsys, V.; Michielssens, S.; Seeliger, D.; De Groot, B. L. Pmx: Automated Protein Structure and Topology Generation for Alchemical Perturbations. *J Comput Chem* **2015**, *36* (5), 348–354. https://doi.org/10.1002/jcc.23804.

(36) Abraham, M. J.; Murtola, T.; Schulz, R.; Páll, S.; Smith, J. C.; Hess, B.; Lindahl, E. GROMACS: High Performance Molecular Simulations through Multi-Level Parallelism from Laptops to Supercomputers. *SoftwareX* **2015**, *1–2*, 19–25. https://doi.org/10.1016/j.softx.2015.06.001.

(37) Davidchack, R. L.; Handel, R.; Tretyakov, M. V. Langevin Thermostat for Rigid Body Dynamics. *The Journal of Chemical Physics* **2009**, *130* (23), 234101. https://doi.org/10.1063/1.3149788.

(38) Parrinello, M.; Rahman, A. Polymorphic Transitions in Single Crystals: A New Molecular Dynamics Method. *Journal of Applied Physics* **1981**, *52* (12), 7182–7190. https://doi.org/10.1063/1.328693.

(39) Hess, B.; Bekker, H.; Berendsen, H. J. C.; Fraaije, J. G. E. M. LINCS: A Linear Constraint Solver for Molecular Simulations. *J. Comput. Chem.* **1997**, *18* (12), 1463–1472. https://doi.org/10.1002/(SICI)1096-987X(199709)18:12<1463::AID-JCC4>3.0.CO;2-H.

(40) Shirts, M. R.; Bair, E.; Hooker, G.; Pande, V. S. Equilibrium Free Energies from Nonequilibrium Measurements Using Maximum-Likelihood Methods. *Phys. Rev. Lett.* **2003**, *91* (14), 140601. https://doi.org/10.1103/PhysRevLett.91.140601.

(41) Crooks, G. E. Entropy Production Fluctuation Theorem and the Nonequilibrium Work Relation for Free Energy Differences. *Phys. Rev. E* **1999**, *60* (3), 2721–2726. https://doi.org/10.1103/PhysRevE.60.2721.
